# Assessment of Risk Factors and Clinical Importance of Enlarged Perivascular Spaces by Whole-Brain Investigation in the Multi-Ethnic Study of Atherosclerosis

**DOI:** 10.1001/jamanetworkopen.2023.9196

**Published:** 2023-04-24

**Authors:** Sokratis Charisis, Tanweer Rashid, Hangfan Liu, Jeffrey B. Ware, Paul N. Jensen, Thomas R. Austin, Karl Li, Elyas Fadaee, Saima Hilal, Christopher Chen, Timothy M. Hughes, Jose Rafael Romero, Jon B. Toledo, Will T. Longstreth, Timothy J. Hohman, Ilya Nasrallah, R. Nick Bryan, Lenore J. Launer, Christos Davatzikos, Sudha Seshadri, Susan R. Heckbert, Mohamad Habes

**Affiliations:** 1 Neuroimage Analytics Laboratory and the Biggs Institute Neuroimaging Core, Glenn Biggs Institute for Alzheimer’s and Neurodegenerative Diseases, University of Texas Health Science Center at San Antonio; 2 Department of Neurology, University of Texas Health Science Center at San Antonio; 3 AI2D Center for AI and Data Science for Integrated Diagnostics, and Center for Biomedical Image Computing and Analytics, University of Pennsylvania, Philadelphia; 4 Department of Radiology, Perelman School of Medicine, University of Pennsylvania, Philadelphia; 5 Department of Medicine, University of Washington, Seattle; 6 Department of Epidemiology, University of Washington, Seattle; 7 Department of Pharmacology, National University of Singapore, Singapore; 8 Memory Aging and Cognition Centre, National University Health System, Singapore; 9 Department of Internal Medicine, Wake Forest School of Medicine, Winston-Salem, North Carolina; 10 Department of Neurology, School of Medicine, Boston University, Boston, Massachusetts; 11 Nantz National Alzheimer Center, Stanley Appel Department of Neurology, Houston Methodist Hospital, Houston, Texas; 12 Department of Neurology, University of Washington, Seattle; 13 Vanderbilt Memory and Alzheimer's Center, Vanderbilt University Medical Center, Nashville, Tennessee; 14 Intramural Research Program, Laboratory of Epidemiology and Population Sciences, National Institute on Aging, National Institutes of Health, Bethesda, Maryland

## Abstract

**Importance:**

Enlarged perivascular spaces (ePVSs) have been associated with cerebral small-vessel disease (cSVD). Although their etiology may differ based on brain location, study of ePVSs has been limited to specific brain regions; therefore, their risk factors and significance remain uncertain.

**Objective:**

Toperform a whole-brain investigation of ePVSs in a large community-based cohort.

**Design, Setting, and Participants:**

This cross-sectional study analyzed data from the Atrial Fibrillation substudy of the population-based Multi-Ethnic Study of Atherosclerosis. Demographic, vascular risk, and cardiovascular disease data were collected from September 2016 to May 2018. Brain magnetic resonance imaging was performed from March 2018 to July 2019. The reported analysis was conducted between August and October 2022. A total of 1026 participants with available brain magnetic resonance imaging data and complete information on demographic characteristics and vascular risk factors were included.

**Main Outcomes and Measures:**

Enlarged perivascular spaces were quantified using a fully automated deep learning algorithm. Quantified ePVS volumes were grouped into 6 anatomic locations: basal ganglia, thalamus, brainstem, frontoparietal, insular, and temporal regions, and were normalized for the respective regional volumes. The association of normalized regional ePVS volumes with demographic characteristics, vascular risk factors, neuroimaging indices, and prevalent cardiovascular disease was explored using generalized linear models.

**Results:**

In the 1026 participants, mean (SD) age was 72 (8) years; 541 (53%) of the participants were women. Basal ganglia ePVS volume was positively associated with age (β = 3.59 × 10^−3^; 95% CI, 2.80 × 10^−3^ to 4.39 × 10^−3^), systolic blood pressure (β = 8.35 × 10^−4^; 95% CI, 5.19 × 10^−4^ to 1.15 × 10^−3^), use of antihypertensives (β = 3.29 × 10^−2^; 95% CI, 1.92 × 10^−2^ to 4.67 × 10^−2^), and negatively associated with Black race (β = −3.34 × 10^−2^; 95% CI, −5.08 × 10^−2^ to −1.59 × 10^−2^). Thalamic ePVS volume was positively associated with age (β = 5.57 × 10^−4^; 95% CI, 2.19 × 10^−4^ to 8.95 × 10^−4^) and use of antihypertensives (β = 1.19 × 10^−2^; 95% CI, 6.02 × 10^−3^ to 1.77 × 10^−2^). Insular region ePVS volume was positively associated with age (β = 1.18 × 10^−3^; 95% CI, 7.98 × 10^−4^ to 1.55 × 10^−3^). Brainstem ePVS volume was smaller in Black than in White participants (β = −5.34 × 10^−3^; 95% CI, −8.26 × 10^−3^ to −2.41 × 10^−3^). Frontoparietal ePVS volume was positively associated with systolic blood pressure (β = 1.14 × 10^−4^; 95% CI, 3.38 × 10^−5^ to 1.95 × 10^−4^) and negatively associated with age (β = −3.38 × 10^−4^; 95% CI, −5.40 × 10^−4^ to −1.36 × 10^−4^). Temporal region ePVS volume was negatively associated with age (β = −1.61 × 10^−2^; 95% CI, −2.14 × 10^−2^ to −1.09 × 10^−2^), as well as Chinese American (β = −2.35 × 10^−1^; 95% CI, −3.83 × 10^−1^ to −8.74 × 10^−2^) and Hispanic ethnicities (β = −1.73 × 10^−1^; 95% CI, −2.96 × 10^−1^ to −4.99 × 10^−2^).

**Conclusions and Relevance:**

In this cross-sectional study of ePVSs in the whole brain, increased ePVS burden in the basal ganglia and thalamus was a surrogate marker for underlying cSVD, highlighting the clinical importance of ePVSs in these locations.

## Key Points

**Question:**

What is the clinical importance of enlarged perivascular spaces detected on brain magnetic resonance imaging?

**Findings:**

This cross-sectional study was conducted in a multiethnic sample of 1026 individuals living in the community. Enlarged perivascular spaces in the basal ganglia and thalamus were associated with magnetic resonance imaging markers of cerebral small-vessel disease.

**Meaning:**

The findings of this study suggest a high burden of enlarged perivascular spaces in the basal ganglia and thalamus may represent underlying vascular brain pathology.

## Teaser

This cross-sectional study examines the association of magnetic resonance imaging–detected enlarged perivascular spaces in several regionals of the brain with clinical outcomes in a multiethnic cohort.

## Introduction

Perivascular spaces (PVSs) are cerebrospinal fluid–filled spaces that surround arteries, arterioles, veins, and venules, following their trajectories from the brain surface into the parenchyma.^1^ Although it is common to see a few enlarged PVSs (ePVSs) in adults, high ePVS burden has been associated with cerebral small-vessel disease (cSVD).^2,3,4,5^ Previous research has revealed associations of ePVSs with hypertension^6^ and with known cSVD imaging phenotypes, such as white matter hyperintensities (WMHs),^5^ and lacunar strokes.^5,7^ However, the clinical importance of ePVSs in the general population and whether they could extend the spectrum of magnetic resonance imaging (MRI) markers of cSVD remain unclear.

A limiting factor in the progress of ePVS research has been the lack of quantitative tools. Most earlier investigations have relied on the construction of manual rating scales,^5,8,9,10,11,12^ using visual inspection of T1- or T2-weighted images^5,8,9,10,11,12^ to assign scores for specific brain regions (most commonly the basal ganglia and centrum semiovale) based on shape, diameter, and approximate ePVS count. These techniques are labor-intensive and impractical for analysis of large-scale epidemiologic data. They also lack quantitative information on individual, region-specific, and total ePVS size; therefore, they might imprecisely reflect the actual ePVS burden. Furthermore, interrater and intrarater variability are important limitations of visual rating methods.^13^

Higher ePVS prevalence has been described in specific brain regions, including the basal ganglia, frontoparietal region, brainstem, and hippocampus.^12^ Studies exploring associations of ePVSs with cSVD have focused primarily on total ePVS burden.^5,9,11,12,14,15,16,17^ However, it has been suggested that ePVSs in different regions may have distinct pathophysiologic characteristics.^18^ Therefore, region-wise analysis of ePVSs might provide deeper insights into their associations with different risk factors and health-related outcomes.

We performed a whole-brain investigation of ePVSs in a large community-based, multiethnic cohort to explore their associations with (1) demographic characteristics and vascular risk factors, (2) MRI markers of brain aging and cSVD, and (3) prevalent cardiovascular disease (CVD). To this end, we used a fully automated deep learning–based method^19,20,21,22^ to identify and study ePVSs in different brain regions, considering their pathogenesis might differ based on brain location.

## Methods

### Participants

The Multi-Ethnic Study of Atherosclerosis (MESA) was designed to investigate subclinical CVD in a diverse population-based sample of 6814 individuals, aged 45 to 84 years at enrollment. The cohort was recruited from 6 field centers across the US.^23^ The baseline examination occurred from 2000 to 2002, and 5 follow-up examinations have been completed since then, including examination 6 from 2016 to 2018. At examination 6, 1942 individuals were invited to participate in the Atrial Fibrillation substudy, which included brain MRI 1 to 2 years later^24^; a total of 1062 participants underwent brain MRI. Of those, 1036 individuals with complete information on demographic characteristics and vascular risk factors were included in the present cross-sectional investigation, conducted from September 2016 to May 2018 (eFigure 1 in Supplement 1). Each study site obtained institutional review board approval, and all participants provided written informed consent. The participants received between $50 and $100 for participation in the main examination (the amount varied from one field center to another), and participants in the Atrial Fibrillation ancillary study received a $50 incentive for completing the Zio Patch cardiac monitor and $50 for completing the brain MRI about 18 months later. This report follows the Strengthening the Reporting of Observational Studies in Epidemiology (STROBE) reporting guideline for cross-sectional studies.

### Brain MRI Acquisition, Processing, and Measures of Interest

Brain MRI scans were acquired (3-Tesla Siemens scanners), and structural sequences included 1-mm isotropic, sagittal, 3-dimensional T1-weighted, T2-weighted, fluid-attenuated inversion recovery (FLAIR), and axial 2-dimensional, 30-direction echo-planar diffusion-tensor imaging. The detailed brain MRI protocol has been described elsewhere.^25^ Measures of interest derived from MRI scans included total intracranial volume, total gray matter volume, total WMH volume, presence of cerebral microbleeds (CMBs), and total white matter fractional anisotropy (WMFA) (eMethods in Supplement 1).

All participant scans were visually inspected for distortions and motion artifacts and were assigned a quality index. Participants with poor-quality scans (n = 10) were excluded from the final analytic sample (eFigure 1 in Supplement 1).

### ePVS Segmentation and Mapping

We adopted our previously developed and tested deep learning model to segment ePVS from T1-weighted, T2-weighted, and FLAIR images (eMethods in Supplement 1).^26^ Considering the focus of previously described clinical rating systems, we quantified ePVSs in the basal ganglia, brainstem, and frontoparietal regions.^12^ Since our automated method can detect ePVSs in the whole brain, we considered the following additional regions to investigate the importance of ePVSs in other brain locations: thalamus, insular region, and temporal region (eMethods, eFigure 2 in Supplement 1). The occipital lobe and cerebellum were not included in the analyses, considering that prevalence of high-burden ePVSs in our sample was relatively low and average model performance was not optimal in these regions (eTable 1 in Supplement 1). Both volumes and counts of ePVSs were quantified for each region. Volumetric data were normalized by dividing the regional ePVS volumes by the total region of interest volume for the respective region.

### Demographic Characteristics, Vascular Risk Factors, and Prevalent CVD

Self-reported age, sex, and race and ethnicity were recorded at the baseline MESA examination. At examination 6, vascular risk factor data were collected: information on smoking status, alcohol consumption, medication use, and intentional physical activity (measured in metabolic equivalents of task per minute per week) were updated, and anthropometric data were measured. Waist-to-hip ratio was calculated by dividing the participant's waist circumference by hip circumference (centimeter/centimeter). Waist-to-hip ratio might be a better estimator of health-related outcomes, including the risk for CVD, stroke, diabetes, and overall mortality, than body mass index.^27,28^ Blood pressure was calculated as the mean of the last 2 of 3 measurements, with the participant resting in a sitting position. Blood glucose (to convert to millimoles per liter, multiply by 0.0555) and hemoglobin A_1c_ (to convert to proportion of total hemoglobin, multiply by 0.01) levels were measured from fasting blood samples. Hyperlipidemia was defined as the use of lipid-lowering medications. Diabetes was defined as reported use of diabetes medications, fasting glucose level 126 mg/dL or greater, or hemoglobin A_1c_ level 6.5% or greater.

After the baseline examination, participants completed telephone follow-up assessments every 9 to 12 months during which they were asked to report any new hospitalizations or diagnoses. Medical records were obtained, and myocardial infarction, heart failure, atrial fibrillation, stroke, and transient ischemic attack (TIA) were ascertained, as previously described.^29^ Prevalent CVD was defined as stroke, TIA, or all-cause CVD (including myocardial infarction, resuscitated cardiac arrest, definite angina or probable angina followed by revascularization, or stroke) that occurred before the date of brain MRI acquisition.

### Statistical Analysis

Normality of data was graphically explored using Q-Q plots and kernel density plots. The variables expressing regional ePVS volumes, WMH volume, and intentional physical activity underwent Tukey ladder of powers transformation^30^ to normalize their distributions.

Initially, we used generalized linear models (gaussian family, identity link function) to examine associations of regional ePVS volumes with demographic characteristics and vascular risk factors. Six models were constructed with the different regional ePVS volumes as the outcomes. Variables were demographic characteristics (age, sex, and race and ethnicity) and vascular risk factors (systolic blood pressure [SBP], use of antihypertensive medications, diabetes, hyperlipidemia, smoking, alcohol consumption, and physical activity). To explore potential associations of regional ePVS volumes with MRI markers of brain aging and cSVD, we constructed a set of generalized linear models (gaussian family, identity link function; unless otherwise stated) with the following MRI indices as outcomes: gray matter volume, WMH volume, presence of CMBs (binomial family, logit link function), WMFA; regional ePVS volumes were the main variables.

Associations of regional ePVS volumes with prevalent CVD were tested using a set of generalized linear models (binomial family, logit link function) with all-cause CVD, stroke, and TIA as outcomes; regional ePVS volumes were the main variables. Subsequent models were further adjusted for vascular risk factors. All models were adjusted for age, sex, race and ethnicity, field center, and intracranial volume.

The following sensitivity analyses were performed. All models were recomputed using regional ePVS counts instead of volumes. The associations of regional ePVS volumes with vascular risk factors were also modeled using quantile regression to explore for potential differential effects based on regional ePVS burden.

Residual plots and generalized variance inflation factors^31^ were inspected for all models. The false discovery rate was controlled at 5% using the Benjamini-Hochberg procedure.^32^ All analyses were performed using R, version 4.2.1 (R Foundation for Statistical Computing, 2022). All statistical tests were unpaired. A 2-sided *P* value ≤.05 was considered statistically significant.

## Results

### Cohort Description and ePVS Spatial Distribution

Participant characteristics (N = 1026) are presented in Table 1. Mean (SD) age was 72 (8) years, 541 participants (53%) were women, and 485 were men (47%). The cohort had a relatively high prevalence of vascular risk factors, including smoking (538 [52%]), alcohol consumption (472 [46%]), diabetes (224 [22%]), use of antihypertensive medications (602 [59%]), and hyperlipidemia (462 [45%]). Mean (SD) waist-to-hip ratio was within the abdominal obesity range for both men (0.97 [0.06]) and women (0.90 [0.08]), according to World Health Organization guidelines (>0.90 for men, >0.85 for women).^33^ The prevalence of all-cause CVD (82 [8%]), stroke (26 [3%]), and TIA (13 [1%]) was relatively low.

**Table 1.** Participant Characteristics (N = 1026)

Characteristic	No. (%)
Age, mean (SD), y	72 (8)
Sex	
Men	485 (47)
Women	541 (53)
Race and ethnicity	
Black, African American	255 (25)
Chinese American	151 (15)
Hispanic	205 (20)
White	415 (40)
Waist-to-hip ratio, mean (SD), cm	
Men	0.97 (0.06)
Women	0.90 (0.08)
Cigarette smoking	
Never	488 (48)
Ever	538 (52)
Current alcohol use	
No	554 (54)
Yes	472 (46)
Total intentional exercise, median (IQR), MET/min/wk	1054 (315-2376)
Systolic blood pressure, mean (SD), mm Hg	127 (21)
Use of antihypertensives	
No	424 (41)
Yes	602 (59)
Diabetes	
No	802 (78)
Yes	224 (22)
Hyperlipidemia	
No	564 (55)
Yes	462 (45)
Prevalent CVD	
All-cause CVD^a^	82 (8)
Stroke	26 (3)
Transient ischemic attack	13 (1)
Brain MRI measures of interest	
Intracranial volume, mean (SD), μL	1 359 451 (145 079)
Total and regional ePVS volumes, median (IQR), as % of total ROI volume	
Total	0.58 (0.42-0.86)
Basal ganglia	1.89 (1.35-2.77)
Thalamus	0.25 (0.14-0.46)
Insular region	0.12 (0.04-0.25)
Brainstem	0.18 (0.11-0.25)
Frontoparietal region	0.58 (0.37-0.91)
Temporal region	0.39 (0.24-0.63)
Total and regional ePVS counts, median (IQR)	
Total	589 (450-754)
Basal ganglia	62 (52-73)
Thalamus	8 (5-11)
Insular region	5 (2-8)
Brainstem	9 (7-11)
Frontoparietal region	389 (292-510)
Temporal region	112 (76-157)
Total gray matter volume, mean (SD), μL	596 759 (64 573)
Total white matter hyperintensitiy volume, median (IQR), μL	3669 (1538-9275)
Cerebral microbleeds	
Present	682 (66)
Absent	344 (34)
Total white matter fractional anisotropy, mean (SD)	0.249 (0.013)

Abbreviations: CVD, cardiovascular disease; ePVS, enlarged perivascular space; MET, metabolic equivalent of task; MRI, magnetic resonance imaging; ROI, region of interest.

^a^ All-cause CVD includes myocardial infarction, resuscitated cardiac arrest, definite angina or probable angina followed by revascularization, or stroke that occurred prior to the date of brain MRI acquisition.

Basal ganglia was the anatomic region with the largest ePVS burden (median, 1.89% of total region-of-interest volume [IQR, 1.35%-2.77%]), followed by (in decreasing order) the frontoparietal region (0.58% [IQR, 0.37%-0.91%]), temporal region (0.39% [IQR, 0.24%-0.63%]), thalamus (0.25% [IQR, 0.14%-0.46%]), brainstem (0.18% [IQR, 0.11%-0.25%]), and insular region (0.12% [IQR, 0.04%-0.25%]). Frequency maps illustrating the spatial distribution of ePVSs in the brain are presented in the Figure. The maps show a clear pattern of lesion clusters, particularly in the deep structures of basal ganglia and thalamus. Region-wise and overall model performance metrics are presented in eTable 1 in Supplement 1.

**Figure.**
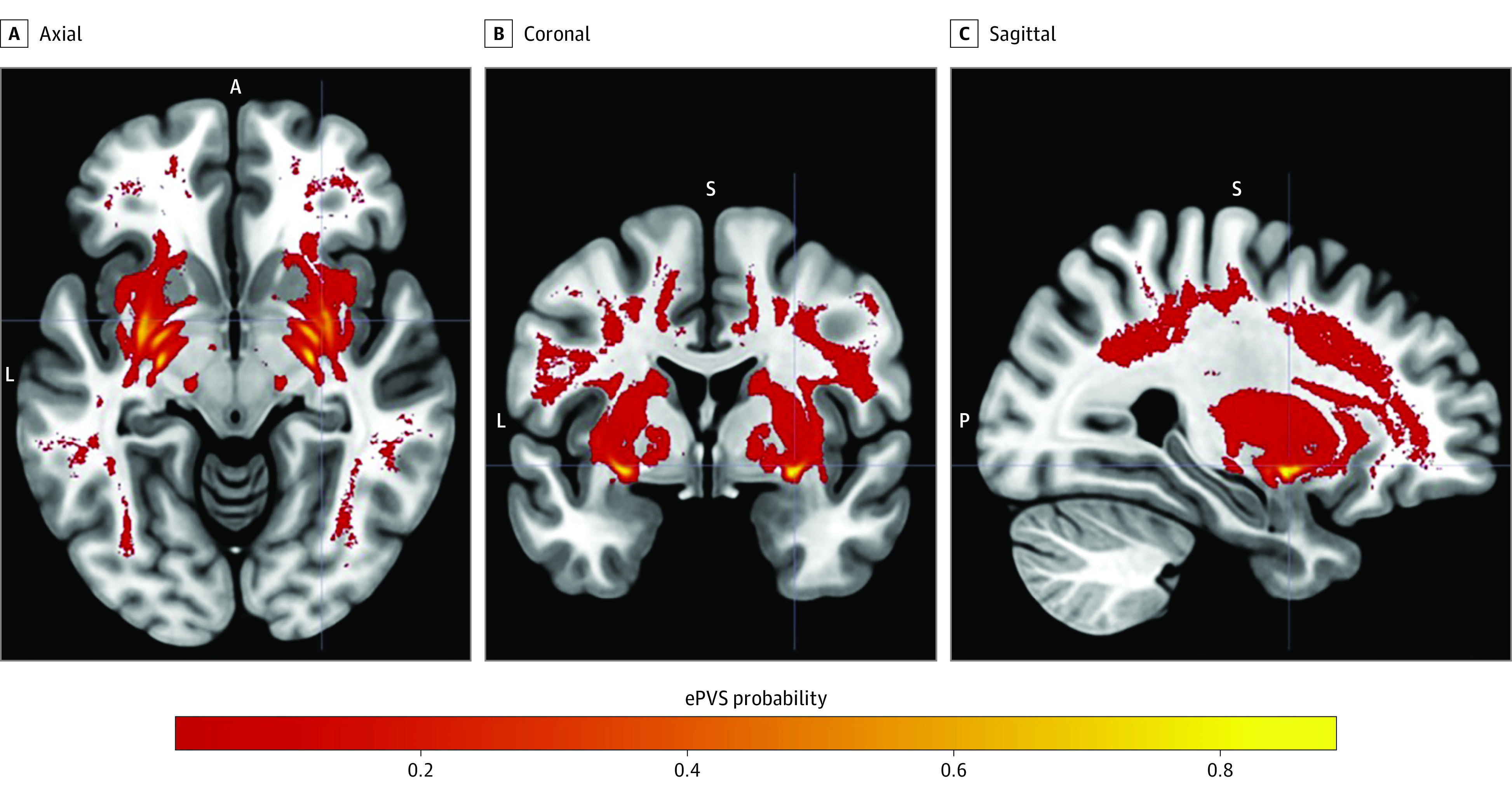
Frequency Maps Depicting the Distribution of Enlarged Perivascular Spaces (ePVSs) Values represent the probability of detecting an ePVS at the respective anatomic location. A indicates anterior; FLAIR, fluid-attenuated inversion recovery; L, left; P, posterior; and S, superior.

### Associations With Demographic and Vascular Risk Factors

Associations of regional ePVS volumes with demographic characteristics (Table 2) and vascular risk factors (Table 3) are presented. Older age was associated with larger basal ganglia (β =  3.59 × 10^−3^; 95% CI, 2.80 × 10^−3^ to 4.39 × 10^−3^), thalamic (β = 5.57 × 10^−4^; 95% CI, 2.19 × 10^−4^ to 8.95 × 10^−4^), and insular (β = 1.18 × 10^−3^; 95% CI, 7.98 × 10^−4^ to 1.55 × 10^−3^) ePVS volumes, and smaller frontoparietal (β = −3.38 × 10^−4^; 95% CI, −5.40 × 10^−4^ to −1.36 × 10^−4^) and temporal (β = −1.61 × 10^−2^; 95% CI, −2.14 × 10^−2^ to −1.09 × 10^−2^) ePVS volumes (eFigure 3 in Supplement 1). Compared with White race, Black race was associated with smaller basal ganglia (β = −3.34 × 10^−2^; 95% CI, −5.08 × 10^−2^ to −1.59 × 10^−2^) and brainstem (β = −5.34 × 10^−3^; 95% CI, −8.26 × 10^−3^ to −2.41 × 10^−3^) ePVS volumes, and Chinese American (β = −2.35 × 10^−1^; 95% CI, −3.83 × 10^−1^ to −8.74 × 10^−2^) and Hispanic (β = −1.73 × 10^−1^; 95% CI, −2.96 × 10^−1^ to −4.99 × 10^−2^) ethnicities were associated with smaller temporal ePVS volume. Systolic blood pressure was positively associated with basal ganglia (β = 8.35 × 10^−4^; 95% CI, 5.19 × 10^−4^ to 1.15 × 10^−3^) and frontoparietal (β = 1.14 × 10^−4^; 95% CI, 3.38 × 10^−5^ to 1.95 × 10^−4^) ePVS volumes, whereas, the use of antihypertensive medications was associated with larger basal ganglia (β = 3.29 × 10^−2^; 95% CI, 1.92 × 10^−2^ to 4.67 × 10^−2^) and thalamic (β = 1.19 × 10^−2^; 95% CI, 6.02 × 10^−3^ to 1.77 × 10^−2^) ePVS volumes.

**Table 2.** Associations of Regional ePVS Volumes With Demographic Characteristics^a^

Demographic characteristic	Anatomic location											
	Basal ganglia, −1 × x^−0.2^		Thalamus, x^0.3^		Insular region, x^0.35^		Brainstem, x^0.425^		Frontoparietal region, x^0.05^		Temporal region, ln(x)	
	β (95% CI)	*P* value	β (95% CI)	*P* value	β (95% CI)	*P* value	β (95% CI)	*P* value	β (95% CI)	*P* value	β (95% CI)	*P* value
Age, per y	3.59 × 10^−3^ (2.80 × 10^−3^ to 4.39 × 10^−3^)	<.001^b^	5.57 × 10^−4^ (2.19 × 10^−4^ to 8.95 × 10^−4^)	.001^b^	1.18 × 10^−3^ (7.98 × 10^−4^ to 1.55 × 10^−3^)	<.001^b^	4.61 × 10^−7^ (−1.33 × 10^−4^ to 1.33 × 10^−4^)	>.99	−3.38 × 10^−4^ (−5.40 × 10^−4^ to −1.36 × 10^−4^)	.001^b^	−1.61 × 10^−2^ (−2.14 × 10^−2^ to −1.09 × 10^−2^)	<.001^b^
Sex												
Women	0 [Reference]		0 [Reference]		0 [Reference]		0 [Reference]		0 [Reference]		0 [Reference]	
Men	−1.68 × 10^−3^ (−1.89 × 10^−2^ to 1.55 × 10^−2^)	.85	−3.16 × 10^−3^ (−1.05 × 10^−2^ to 4.16 × 10^−3^)	.40	−3.58 × 10^−3^ (−1.17 × 10^−2^ to 4.58 × 10^−3^)	.39	−1.52 × 10^−3^ (−4.40 × 10^−3^ to 1.35 × 10^−3^)	.30	−4.19 × 10^−3^ (−8.56 × 10^−3^ to 1.76 × 10^−4^)	.06	−1.55 × 10^−1^ (−2.69 × 10^−1^ to −4.06 × 10^−2^)	.01
Race and ethnicity												
Black, African American	−3.34 × 10^−2^ (−5.08 × 10^−2^ to −1.59 × 10^−2^)	<.001^b^	3.64 × 10^−3^ (−3.80 × 10^−3^ to 1.11 × 10^−2^)	.34	4.78 × 10^−4^ (−7.82 × 10^−3^ to 8.78 × 10^−3^)	.91	−5.34 × 10^−3^ (−8.26 × 10^−3^ to −2.41 × 10^−3^)	<.001^b^	−8.51 × 10^−4^ (−5.30 × 10^−3^ to 3.59 × 10^−3^)	.71	−6.51 × 10^−2^ (−1.81 × 10^−1^ to 5.11 × 10^−2^)	.27
Chinese American	−1.68 × 10^−2^ (−3.91 × 10^−2^ to 5.43 × 10^−3^)	.14	−5.24 × 10^−3^ (−1.47 × 10^−2^ to 4.23 × 10^−3^)	.28	−3.67 × 10^−3^ (−1.42 × 10^−2^ to 6.90 × 10^−3^)	.50	−3.67 × 10^−3^ (−7.39 × 10^−3^ to 6.14 × 10^−5^)	.05	−6.50 × 10^−3^ (−1.22 × 10^−2^ to −8.40 × 10^−4^)	.02	−2.35 × 10^−1^ (−3.83 × 10^−1^ to −8.74 × 10^−2^)	.002^b^
Hispanic	−2.05 × 10^−2^ (−3.90 × 10^−2^ to −1.92 × 10^−3^)	.03	−8.92 × 10^−3^ (−1.68 × 10^−2^ to −1.02 × 10^−3^)	.03	6.39 × 10^−3^ (−2.41 × 10^−3^ to 1.52 × 10^−2^)	.16	−3.13 × 10^−3^ (−6.24 × 10^−3^ to −2.32 × 10^−5^)	.05	−4.10 × 10^−3^ (−8.82 × 10^−3^ to 6.17 × 10^−4^)	.09	−1.73 × 10^−1^ (−2.96 × 10^−1^ to −4.99 × 10^−2^)	.006^b^
White	0 [Reference]		0 [Reference]		0 [Reference]		0 [Reference]		0 [Reference]		0 [Reference]	

Abbreviation: ePVS, enlarged perivascular space.

^a^ Values represent β coefficients, reflecting the change in the transformed response variable for 1-increment increase in the variable and their 95% CIs and raw *P* values. Results from generalized linear models with regional ePVS volumes as the outcomes, and demographic characteristics as variables; models were adjusted for field center, intracranial volume, and vascular risk factors (systolic blood pressure, use of antihypertensive medications, diabetes, hyperlipidemia, smoking, alcohol consumption, waist-to-hip ratio, and physical activity). Regional ePVS volumes have undergone the Tukey ladder of powers transformation.

^b^ Significant after false-discovery rate control at 5% using the Benjamini-Hochberg procedure.

**Table 3.** Associations of Regional ePVS Volumes With Vascular Risk Factors^a^

Vascular risk factor	Anatomic location											
	Basal ganglia, −1 × x^−0.125^		Thalamus, x^0.325^		Insular region, x^0.35^		Brainstem, x^0.45^		Frontoparietal region, x^0.05^		Temporal region, ln(x)	
	β (95% CI)	*P* value	β (95% CI)	*P* value	β (95% CI)	*P* value	β (95% CI)	*P* value	β (95% CI)	*P* value	β (95% CI)	*P* value
SBP, per mm Hg	8.35 × 10^−4^ (5.19 × 10^−4^ to 1.15 × 10^−3^)	<.001^^b^^	1.60 × 10^−4^ (2.54 × 10^−5^ to 2.95 × 10^−4^)	.02	1.28 × 10^−5^ (−1.37 × 10^−4^ to 1.63 × 10^−4^)	.87	7.84 × 10−6 (−4.51 × 10^−5^ to 6.08 × 10^−5^)	.77	1.14 × 10^−4^ (3.38 × 10^−5^ to 1.95 × 10^−4^)	.005^^b^^	2.69 × 10^−3^ (5.85 × 10^−4^ to 4.79 × 10^−3^)	.01
Use of antihypertensives	3.29 × 10^-2^ (1.92 × 10^−2^ to 4.67 × 10^−2^)	<.001	1.19 × 10^−2^ (6.02 × 10^−3^ to 1.77 × 10^−2^)	<.001^^b^^	2.77 × 10^−5^ (−6.51 × 10^−3^ to 6.57 × 10^−3^)	.99	1.96 × 10^−3^ (−3.43 × 10^−4^ to 4.27 × 10^−3^)	.10	9.96 × 10^−4^ (−2.51 × 10^−3^ to 4.50 × 10^−3^)	.58	5.01 × 10^−2^ (−4.14 × 10^−2^ to 1.42 × 10^−1^)	.28
Diabetes	−1.52 × 10^−3^ (−1.72 × 10^−2^ to 1.41 × 10^−2^)	.85	−4.65 × 10^−3^ (−1.13 × 10^−2^ to 2.02 × 10^−3^)	.17	7.93 × 10^−3^ (4.91 × 10^−4^ to 1.54 × 10^−2^)	.04	−3.21 × 10^−4^ (−2.94 × 10^−3^ to 2.30 × 10^−3^)	.81	4.90 × 10^−3^ (9.20 × 10^−4^ to 8.89 × 10^−3^)	.02	1.25 × 10^−1^ (2.13 × 10^−2^ to 2.30 × 10^−1^)	.02
Hyperlipidemia	6.62 × 10^−3^ (−6.59 × 10^−3^ to 1.98 × 10^−2^)	.33	1.22 × 10^−3^ (−4.41 × 10^−3^ to 6.84 × 10^−3^)	.67	−6.52 × 10^−3^ (−1.28 × 10^−2^ to −2.42 × 10^−4^)	.04	−7.36 × 10^−4^ (−2.95 × 10^−3^ to 1.48 × 10^−3^)	.52	−2.75 × 10^−3^ (−6.11 × 10^−3^ to 6.16 × 10^−4^)	.11	−4.42 × 10^−2^ (−1.32 × 10^−1^ to 4.37 × 10^−2^)	.33
Smoking	−3.69 × 10^−3^ (−1.63 × 10^−2^ to 8.89 × 10^−3^)	.57	−5.06 × 10^−4^ (−5.87 × 10^−3^ to 4.86 × 10^−3^)	.85	4.79 × 10^−5^ (−5.93 × 10^−3^ to 6.03 × 10^−3^)	.99	1.62 × 10^−4^ (−1.95 × 10^−3^ to 2.27 × 10^−3^)	.88	−2.17 × 10^−3^ (−5.37 × 10^−3^ to 1.04 × 10^−3^)	.19	−8.04 × 10^−2^ (−1.64 × 10^−1^ to 3.24 × 10^−3^)	.06
Alcohol	−7.01 × 10^−3^ (−2.03 × 10^−2^ to 6.27 × 10^−3^)	.30	1.18 × 10^−3^ (−4.48 × 10^−3^ to 6.84 × 10^−3^)	.68	−2.12 × 10^−3^ (−8.43 × 10^−3^ to 4.19 × 10^−3^)	.51	1.41 × 10^−3^ (−8.11 × 10^−4^ to 3.64 × 10^−3^)	.21	3.20 × 10^−4^ (^−3^.06 × 10^−3^ to 3.70 × 10^−3^)	.85	−2.12 × 10^−2^ (−1.10 × 10^−1^ to 6.71 × 10^−2^)	.64
WHR	7.90 × 10^−2^ (−1.23 × 10^−2^ to 1.70 × 10^−1^)	.09	−2.40 × 10^−2^ (−6.29 × 10^−2^ to 1.49 × 10^−2^)	.23	−1.20 × 10^−2^ (−5.54 × 10^−2^ to 3.14 × 10^−2^)	.59	−2.00 × 10^−2^ (−3.53 × 10^−2^ to −4.74 × 10^−3^)	.01	−2.42 × 10^−2^ (−4.75 × 10^−2^ to −9.79 × 10^−4^)	.04	−7.08 × 10^−1^ (−1.32 to −1.01 × 10 )	.02
Physical activity, x0.375	−3.79 × 10^−5^ (−7.53 × 10^−4^ to 6.78 × 10^−4^)	.92	−4.19 × 10−6 (−3.09 × 10^−4^ to 3.01 × 10^−4^)	.98	3.28 × 10^−5^ (−3.07 × 10^−4^ to 3.73 × 10^−4^)	.85	−5.05 × 10^−5^ (−1.70 × 10^−4^ to 6.94 × 10^−5^)	.41	−8.22 × 10^−5^ (−2.64 × 10^−4^ to 9.99 × 10^−5^)	.38	6.03 × 10^−4^ (−4.15 × 10^−3^ to 5.36 × 10^−3^)	.8

Abbreviations: ePVS, enlarged perivascular space; MET, metabolic equivalent of task; SBP, systolic blood pressure; WHR, waist-to-hip ratio.

^a^ Results from generalized linear models with regional ePVS volumes as the outcomes, and vascular risk factors as variables; models were adjusted for age, sex, race and ethnicity, field center, and total intracranial volume. Regional ePVS volumes and intentional physical activity (in MET/min/week) have undergone the Tukey ladder of powers transformation. Values represent β coefficients, reflecting the change in the transformed response variable for 1-increment increase in the variable and their 95% CIs and raw *P* values.

^b^ Significant after false-discovery rate control at 5% using the Benjamini-Hochberg procedure.

### Associations With MRI Markers of Brain Aging and cSVD

Associations between regional ePVS volumes and structural MRI indices are presented in Table 4. Larger basal ganglia and thalamic ePVS volumes were associated with larger total WMH volume (basal ganglia: β = 3.32 × 10^−1^; 95% CI, 2.83 × 10^−1^ to 3.82 × 10^−1^ and thalamic: β = 4.50 × 10^−1^; 95% CI, 3.24 × 10^−1^ to 5.75 × 10^−1^), lower WMFA (basal ganglia: β = −5.42 × 10^−2^; 95% CI, −6.74 × 10^−2^ to −4.11 × 10^−2^ and thalamic: β = −7.04 × 10^−2^; 95% CI, −1.03 × 10^−1^ to −3.80 × 10^−2^), and higher odds for CMBs (basal ganglia: odds ratio [OR], 1.08 × 10^1^; 95% CI, 2.85 to 4.19 × 10^1^ and thalamic: OR, 1.62 × 10^2^; 95% CI, 6.75 to 4.05 × 10^3^). Larger insular ePVS volume was associated with smaller total WMH volume (β = −2.06 × 10^−1^; 95% CI, −3.21 × 10^−1^ to −9.01 × 10^−2^). Temporal ePVS volume was positively associated with total gray matter volume (β = 4.80 × 10^3^; 95% CI, 2.12 × 10^3^ to 7.49 × 10^3^).

**Table 4.** Associations of Regional ePVS Volumes With Structural MRI Indices^a^

Structural MRI index	Anatomic location											
	Basal ganglia, −1 × x−0.2		Thalamus, x0.3		Insular region, x0.35		Brainstem, x0.425		Frontoparietal region, x0.05		Temporal region, ln(x)	
	β (95% CI)	P value	β (95% CI)	P value	β (95% CI)	P value	β (95% CI)	P value	β (95% CI)	P value	β (95% CI)	P value
Total gray matter volume	−8.30 × 10^3^ (−2.58 × 10^4^ to 9.21 × 10^3^)	.35	2.87 × 10^4^ (−1.34 × 10^4^ to 7.07 × 10^4^)	.18	−4.83 × 10^4^ (−8.62 × 10^4^ to −1.0^3^ × 10^4^)	.01	9.07 × 10^4^ (−1.70 × 10^4^ to 1.98 × 10^5^)	.10	8.04 × 10^4^ (9.99 × 10^3^ to 1.51 × 10^5^)	.03	4.80 × 10^3^ (2.12 × 10^3^ to 7.49 × 10^3^)	<.001^^b^^
Total WMH volume, x0.025	3.32 × 10^−1^ (2.83 × 10^−1^ to 3.82 × 10^−1^)	<.001^^b^^	4.50 × 10^−1^ (3.24 × 10^−1^ to 5.75 × 10^−1^)	<.001^^b^^	−2.06 × 10^−1^ (−3.21 × 10^−1^ to −9.01 × 10^−2^)	<.001^^b^^	1.18 × 10^−1^ (−2.14 × 10^−1^ to 4.49 × 10^−1^)	.49	−2.28 × 10^−1^ (−4.43 × 10^−1^ to −1.34 × 10^−2^)	.04	−5.42 × 10^−3^ (−1.37 × 10^−2^ to 2.82 × 10^−3^)	.20
Presence of CMBs, OR	1.08 × 10^1^ (2.85 to 4.19 × 10^1^)	<.001^^b^^	1.62 × 102 (6.75 to 4.05 × 10^3^)	.002^^b^^	7.65 × 10^−1^ (4.40 × 10^−2^ to 1.33 × 10^1^)	.85	1.67 × 10^3^ (4.95 × 10^−1^ to 6.14 × 106)	.07	7.19 × 10^1^ (3.60 × 10^−1^ to 1.49 × 10^4^)	.12	1.19 (9.68 × 10^−1^ to 1.45)	.10
Total WM fractional anisotropy	−5.42 × 10^−2^ (−6.74 × 10^−2^ to −4.11 × 10^−2^)	<.001^^b^^	−7.04 × 10^−2^ (−1.03 × 10^−1^ to −3.80 × 10^−2^)	<.001^^b^^	−3.19 × 10^−2^ (−6.16 × 10^−2^ to −2.29 × 10^−3^)	.04	−4.89 × 10^−2^ (−1.33 × 10^−1^ to 3.47 × 10^−2^)	.25	−7.19 × 10^−2^ (−1.27 × 10^−1^ to −1.72 × 10^−2^)	.01	−2.45 × 10^−3^ (−4.54 × 10^−3^ to −3.55 × 10^−4^)	.02

Abbreviations: CMBs, cerebral microbleeds; ePVS, enlarged perivascular space; MRI, magnetic resonance imaging; OR, odds ratio; WM, white matter; WMH, white matter hyperintensity.

^a^ Results from generalized linear models with structural MRI indices as the outcomes, and regional ePVS volumes as variables; models were adjusted for age, sex, race and ethnicity, field center, and intracranial volume. Regional ePVS volumes and total WMH volume have undergone the Tukey ladder of powers transformation. Values represent β coefficients, reflecting the change in the response variable for 1-increment increase in the transformed variable, and their respective 95% CIs and raw *P* values.

^b^ Significant after false-discovery rate control at 5% using the Benjamini-Hochberg procedure.

### Associations With Prevalent CVD

Associations of regional ePVS volumes with prevalent CVD are presented in eTable 2 in Supplement 1. Larger basal ganglia and thalamic ePVS volumes were associated with higher odds for all-cause CVD (basal ganglia: OR, 80.7; 95% CI, 7.61-901 and thalamus: OR, 4960; 95% CI, 23.6-105 × 10^4^); these findings were not significant after adjustment for vascular risk factors. Larger thalamic ePVS volume was associated with higher odds for TIA, even after adjustment for vascular risk factors (OR, 174 × 10^6^; 95% CI, 493-984 × 10^11^).

### Sensitivity Analyses

Models with regional ePVS counts revealed similar findings (eTables 3-6 in Supplement 1). Compared with the original models, quantile regression models at the 0.25, 0.50, and 0.75 quantiles of regional ePVS volumes revealed that the observed associations of regional ePVS volumes with vascular risk factors were stronger for higher regional ePVS burden (eTables 7-9 in Supplement 1). For high regional ePVS burden, the presence of diabetes was associated with larger frontoparietal and temporal ePVS volumes.

## Discussion

In the present study, we leveraged a deep learning–based method to quantify ePVSs in the whole brain and identified distinctive differential associations of deep brain ePVSs compared with other anatomic locations. The observed heterogeneity in prevalence and clinical associations could be partially explained by different pathophysiologic substrates leading to the appearance of these lesions.

### Spatial ePVS Patterns and Associations With Demographic and Vascular Risk Factors

In concordance with earlier evidence, the highest ePVS burden was detected in the basal ganglia, frontoparietal, and temporal regions.^12,34,35^ Additionally, we identified ePVSs of relatively lower burden in the thalamic, brainstem, and insular regions. The present results replicate previous findings suggesting a positive association between hypertension and basal ganglia ePVSs^6,36,37,38^ and expand their generalizability to a racially and ethnically diverse population. A positive association between SBP and frontoparietal ePVSs was also found, even though such an association was present in some,^6,35,38^ but not all, previously analyzed population samples.^34,37^ Furthermore, although the comparison between thalamic ePVSs and SBP was not significant after correction for multiple testing, there was an association between thalamic ePVSs and the use of antihypertensive medications. Contrary to other studies,^6,34,35,36,37^ we also observed positive associations between ePVS volumes and diabetes in the frontoparietal and temporal regions for high regional ePVS burdens.

Our findings corroborate earlier analyses that have consistently noted increased basal ganglia ePVSs with age.^6,34,35,36,37,39^ However, previous findings regarding the association of age with ePVSs in other brain regions have been conflicting, with most investigations reporting no association of age with white matter,^34,35,37,40^ brainstem,^34^ and temporal region ePVSs.^34,35^ In contrast, in this study, older age was associated with larger thalamic and insular ePVS volumes, and also with smaller frontoparietal and temporal ePVS volumes. Similarly, the results of earlier research on the association of ePVSs in different regions with sex have been mixed.^6,35,37^ In our sample, sex was not associated with regional ePVS volumes. There are very limited data on the associations of ePVSs with race and ethnicity. To our knowledge, the present study is the first to investigate this factor in a multiethnic population and report higher ePVS burden in White participants, compared with other racial and ethnic groups.

Apart from potential differences in the populations studied, there are various other possible explanations for the apparent inconsistencies surrounding the findings of earlier research on ePVS risk factors. The different methods used for ePVS quantification could contribute to the variability of previous findings, as most approaches relied on manual ePVS rating.^6,35,37,38^ In visual ePVS scoring methods, the rating is based on a single axial MRI slice, whereas automated segmentation quantifies ePVSs across the entire region of interest volume; therefore, automated scores may be more robust than manual scales since they are less sensitive to perturbations related to missed or heterogeneous lesions.^36,41^ Furthermore, intracranial volume appears to be associated with larger PVSs, as people with larger intracranial volumes may need larger blood vessels to maintain brain blood supply^42^; this might subsequently allow for greater PVS dilation.^36^ However, in some studies, the associations between regional ePVS volumes and risk factors were not adjusted for intracranial volume, which might lead to residual confounding.^34,35,37,38^ In addition, almost all earlier analyses were based solely on lesion count for ePVS quantification.^6,34,35,37,38,39,40^ In this approach, we used regional ePVS volumetric analysis, considering that it might better reflect underlying lesion burden. Sensitivity analysis with regional ePVS counts revealed similar findings.

### Associations With MRI Markers of Brain Aging and cSVD

The study of ePVSs in association with other cSVD neuroimaging biomarkers, such as WMH and CMBs, has been mostly confined to the basal ganglia and frontoparietal regions, due to limitations imposed by the use of manual rating methods.^39,40,43,44,45^ In this work, we present a whole-brain investigation, and the associations noted appear to primarily involve basal ganglia and thalamic ePVS burden. Similar associations were noted with total WMFA, a marker of myelination and white matter microstructural integrity,^46^ which has been associated with cSVD and cognitive impairment.^47,48,49^ To our knowledge, this is the first study exploring these associations; therefore, the present findings need to be ascertained by future research.

Important to consider is the topographic distribution of the above-mentioned associations, as ePVSs in different brain regions may be the result of distinct underlying pathogenetic processes.^14,18,43,50^ The present results suggest that ePVS burden in the basal ganglia and thalamus increases with age and is associated with SBP and/or the use of antihypertensive medications, as well as with other MRI markers of vascular brain injury. Taken together, these findings suggest that increased deep brain ePVS burden is more likely to represent underlying vascular pathology than a normal variant.

Perivascular space is the anatomic basis of the glymphatic system, a paravascular pathway crucial for removing cerebral waste products and maintaining brain homeostasis.^18,51^ Perivascular space enlargement might reflect glymphatic system dysfunction,^18^ a potentially important contributor to cSVD abnormalities.^50^ Although the exact mechanisms remain unknown, an imbalance between cerebrospinal fluid inflow and outflow might be responsible for PVS enlargement.^18^ Basal ganglia is an anatomic region especially vulnerable to disruptions in the steady flow state of the PVS system, since the largest cerebrospinal fluid influxes occur along the large ventral perforating arteries of the basal ganglia.^50^ The fact that this region is a common site of ePVS formation in cSVD^50^ further supports that ePVSs might be linked to cSVD through glymphatic dysfunction caused by regional disruptions in flow dynamics.

The positive association of temporal ePVS volume with total gray matter volume and the negative association between insular ePVS volume and total WMH volume were more puzzling to interpret. Due to the physical proximity of ePVSs to vascular structures, the ePVS segmentation algorithm might exhibit increased rates of false-positive findings in certain brain areas with large vasculature. Indeed, region-wise evaluation of model performance revealed relatively a lower performance in the temporal and insular regions, so the replicability and validity of findings pertaining to these regions must be ascertained by future research.

### Associations With Prevalent CVD

Enlarged PVSs in general,^5,6,52^ and in basal ganglia in particular,^6,34,35^ have been associated with stroke. Although we did not find an association with ischemic stroke, larger thalamic ePVS volume was associated with higher odds for TIA, even after adjustment for vascular risk factors. Since the MESA study was originally developed to investigate subclinical CVD, our cohort had an expectedly low prevalence of CVD, which might have led to insufficient power to detect potential associations with prevalent stroke. Therefore, such associations cannot be excluded and need to be further explored, especially considering earlier evidence pointing toward a positive association.

### Limitations

This study has limitations. While the method used for ePVS segmentation presents substantial advantages over previously used manual rating methods, additional model training might be required to improve its generalizability for application in different cohorts. Furthermore, the cross-sectional design of the present analysis does not allow for derivation of temporal associations. Although the temporality and directionality of the reported findings might not be relevant for associations with demographic characteristics, these need to be ascertained for associations pertaining to vascular risk factors, other imaging biomarkers of cSVD, and CVD. For example, it is critical to elucidate whether hypertension predates the development of ePVSs (as the opposite would indicate residual confounding since it is unlikely for ePVSs to cause hypertension) and whether the development of ePVSs in specific brain regions antedates or follows other structural changes detected on brain MRI or vascular incidents. Other potential limitations include the cohort's low prevalence of CVD, which might have led to insufficient power to capture potential associations with these outcomes.

## Conclusions

The present findings provide important insights into the clinical importance of ePVSs in different brain regions. In addition, the results highlight the potential utility of ePVSs in the basal ganglia and thalamus as surrogate imaging biomarkers of vascular brain injury.

## Notes

Supplement 1. **eMethods.** Specific Methods
eReferences
**eTable 1.** Overall and Region-Wise Average Model Performance Metrics Comparing the Model Predicted Counts and Volumes of ePVS Lesions to the Ground Truth **eTable 2.** Associations of Regional ePVS Volumes With Prevalent Cardiovascular Disease **eTable 3.** Associations of Regional ePVS Counts With Demographic Characteristics **eTable 4.** Associations of Regional ePVS Counts With Vascular Risk Factors **eTable 5.** Associations of Regional ePVS Counts With Structural MRI Indices **eTable 6.** Associations of Regional ePVS Counts With Prevalent Cardiovascular Disease **eTable 7.** Associations of Regional ePVS Counts With Vascular Risk Factors for Low Regional ePVS Volumes (at the 25th Percentile) **eTable 8.** Associations of Regional ePVS Volumes With Vascular Risk Factors for Median Regional ePVS Volumes (at the 50th Percentile) **eTable 9.** Associations of Regional ePVS Volumes With Vascular Risk Factors for High Regional ePVS Volumes (at the 75th Percentile) **eFigure 1.** Flowchart of Study Sample **eFigure 2.** Anatomic Regions Based on the Existing MUSE Segmentations **eFigure 3.** Predicted Normalized Regional ePVS Volumes (y-Axis) Over Age (x-Axis)

Supplement 2.
Data Sharing Statement
