# Supplementary material for: Assessment of Risk Factors and Clinical Importance of Enlarged Perivascular Spaces by Whole-Brain Investigation in the Multi-Ethnic Study of Atherosclerosis
Source: JAMA Netw Open. 2023 Apr 24;6(4):e239196. doi: 10.1001/jamanetworkopen.2023.9196 (PMC10126873; doi:10.1001/jamanetworkopen.2023.9196)
Supplement: Supplement 1. — eMethods. Specific Methods eReferences eTable 1. Overall and Region-Wise Average Model Performance Metrics Comparing the Model Predicted Counts and Volumes of ePVS Lesions to the Ground Truth eTable 2. Associations of Regional ePVS Volumes With Prevalent Cardiovascular Disease eTable 3. Associations of Regional ePVS Counts With Demographic Characteristics eTable 4. Associations of Regional ePVS Counts With Vascular Risk Factors eTable 5. Associations of Regional ePVS Counts With Structural MRI Indices eTable 6. Associations of Regional ePVS Counts With Prevalent Cardiovascular Disease eTable 7. Associations of Regional ePVS Counts With Vascular Risk Factors for Low Regional ePVS Volumes (at the 25th Percentile) eTable 8. Associations of Regional ePVS Volumes With Vascular Risk Factors for Median Regional ePVS Volumes (at the 50th Percentile) eTable 9. Associations of Regional ePVS Volumes With Vascular Risk Factors for High Regional ePVS Volumes (at the 75th Percentile) eFigure 1. Flowchart of Study Sample eFigure 2. Anatomic Regions Based on the Existing MUSE Segmentations eFigure 3. Predicted Normalized Regional ePVS Volumes (y-Axis) Over Age (x-Axis) [file jamanetwopen-e239196-s001.pdf]

## Supplementary Online Content

Charisis S, Rashid T, Liu H, et al. Assessment of risk factors and clinical importance of enlarged perivascular spaces by whole-brain investigation in the Multi-Ethnic Study of Atherosclerosis. *JAMA Netw Open*. 2023;6(4):e239196. doi:10.1001/jamanetworkopen.2023.9196

**eMethods.** Specific Methods

**eReferences**

**eTable 1.** Overall and Region-Wise Average Model Performance Metrics Comparing the Model Predicted Counts and Volumes of ePVS Lesions to the Ground Truth

**eTable 2.** Associations of Regional ePVS Volumes With Prevalent Cardiovascular Disease

**eTable 3.** Associations of Regional ePVS Counts With Demographic Characteristics

**eTable 4.** Associations of Regional ePVS Counts With Vascular Risk Factors

**eTable 5.** Associations of Regional ePVS Counts With Structural MRI Indices

**eTable 6.** Associations of Regional ePVS Counts With Prevalent Cardiovascular Disease

**eTable 7.** Associations of Regional ePVS Counts With Vascular Risk Factors for Low Regional ePVS Volumes (at the 25th Percentile)

**eTable 8.** Associations of Regional ePVS Volumes With Vascular Risk Factors for Median Regional ePVS Volumes (at the 50th Percentile)

**eTable 9.** Associations of Regional ePVS Volumes With Vascular Risk Factors for High Regional ePVS Volumes (at the 75th Percentile)

**eFigure 1.** Flowchart of Study Sample

**eFigure 2.** Anatomic Regions Based on the Existing MUSE Segmentations

**eFigure 3.** Predicted Normalized Regional ePVS Volumes (y-Axis) Over Age (x-Axis)

This supplementary material has been provided by the authors to give readers additional information about their work.

© 2023 Charisis S,et al. *JAMA Network Open.*

## eMethods. Specific Methods

### Derivation of MRI measures of interest

The present investigation included data from T1w, T2w, FLAIR, and DTI MRI sequences. An automated pipeline was applied to preprocess structural MRI data, which included inhomogeneity correction[1] and extraction of the intracranial brain tissues and cerebrospinal fluid using multi-atlas skull-stripping.[2] Anatomical regions of interest (ROIs) were identified using a multi-atlas label fusion method[3] that was used to segment gray matter (GM) and white matter (WM) tissues. ICV was the sum of GM, WM, and cerebrospinal fluid. The volume of WMH was measured from inhomogeneity-corrected and co-registered FLAIR and T1w images using a deep learning-based segmentation method.[4] CMB segmentation was performed as previously described.[5] FA is a scalar measure of WM integrity calculated from DTI using automated pipelines.[6] FA is the degree to which water diffusion is limited to a single dimension and is a scalar ranging from 0, indicating equivalent motion in all directions, to 1, indicating motion restricted to a single direction. Low FA indicates poor WM integrity and is a measure of WM injury burden in diseases affecting the WM, including cSVD.

### Deep learning model for ePVS segmentation

For the purposes of the present investigation, ePVS were segmented and quantified using the deep learning method described elsewhere.[7] Briefly, the model is based on the U-Net architecture,[8, 9] and consists of a series of down-sampling convolution blocks followed by an equal number of up-sampling convolution blocks. The model takes multi-channel data (co-registered T1w, T2w, and FLAIR MRI data) as input and generate a multiclass segmentation map of the lesion(s) of interest. Initially, ePVS for 21 randomly selected participants were manually labeled by an expert neuro-radiologist (JBW), using co-registered T1w, T2w and FLAIR MRI data. This labeled dataset was used as the ground truth for model training. The MRI data and ground truth were augmented with combinations of geometric transforms such as flip-up or -down, flip-left or -right, translations and rotations, to increase the size of the training dataset. Model training was performed using leave-one-out cross-validation where 1 participant's data is reserved for testing (or model evaluation) while the remaining 20 participants' data are used for model training and within-training validation. Once training is complete, the model's performance is evaluated with the reserved participant's data. This process was repeated for each participant, thereby ensuring that the maximal amount of the available data was used for model training. The performance of the deep learning method was evaluated using the pooled segmentations of all 21 trained models. Performance was evaluated based on a number of metrics, including lesion-wise sensitivity and precision, which were 82% and 83%, respectively.

### Region-wise mapping of ePVS

In this investigation, the brain was parcellated into a number of regions-of-interest (ROI), similar to the mapping described in previous report.[7] The ROI-based mapping of individual ePVS regions was as follows: (i) basal ganglia (included ROIs: caudate, internal capsule, putamen, globus pallidum), (ii) thalamus (included ROI: thalamus), (iii) insular region (included ROIs: anterior and posterior insula), (iv) brainstem (included ROIs: ventral diencephalon, brainstem), (v) frontoparietal region (included ROIs: frontal lobe WM, parietal lobe WM, corpus callosum), (vi) temporal region (included ROIs: hippocampus, temporal lobe WM).

© 2023 Charisis S,et al. *JAMA Network Open.*

## eReferences


1. Tustison NJ, Avants BB, Cook PA, et al. N4ITK: Improved N3 Bias Correction. *IEEE Transactions on Medical Imaging*. 2010;29(6):1310-1320. doi:10.1109/TMI.2010.2046908
2. Doshi J, Erus G, Ou Y, et al. Multi-atlas skull-stripping. *Academic radiology*. 2013;20(12):1566-1576.
3. Doshi J, Erus G, Ou Y, et al. MUSE: MUlti-atlas region Segmentation utilizing Ensembles of registration algorithms and parameters, and locally optimal atlas selection. *Neuroimage*. 2016;127:186-195.
4. Doshi J, Erus G, Habes M, et al. DeepMRSeg: A convolutional deep neural network for anatomy and abnormality segmentation on MR images. *arXiv preprint arXiv:190702110*. 2019;
5. Rashid T, Abdulkadir A, Nasrallah IM, et al. DEEPMIR: a deep neural network for differential detection of cerebral microbleeds and iron deposits in MRI. *Sci Rep*. Jul 8 2021;11(1):14124. doi:10.1038/s41598-021-93427-x
6. Haight T, Bryan RN, Erus G, et al. White matter microstructure, white matter lesions, and hypertension: An examination of early surrogate markers of vascular-related brain change in midlife. *NeuroImage: Clinical*. 2018;18:753-761.
7. Rashid T, Liu H, Ware JB, et al. Deep Learning Based Detection of Enlarged Perivascular Spaces on Brain MRI. *NeuroImage: Reports*. 2023;In Press
8. Çiçek Ö, Abdulkadir A, Lienkamp SS, et al. 3D U-Net: learning dense volumetric segmentation from sparse annotation. Springer; 2016:424-432.
9. Ronneberger O, Fischer P, Brox T. U-net: Convolutional networks for biomedical image segmentation. Springer; 2015:234-241.


© 2023 Charisis S,et al. *JAMA Network Open.*

**eTable 1: Overall and region-wise average model performance metrics comparing the model predicted counts and volumes of ePVS lesions to the ground truth.**

| | Sensitivity | Precision | Magnitude Accuracy | Volumetric Similarity | Area Under the Curve |
|---|---|---|---|---|---|
| Overall | 0.82 | 0.84 | 1.17 | 0.82 | 0.74 |
| Basal Ganglia | 0.92 | 0.89 | 1.28 | 0.92 | 0.82 |
| Thalamus | 0.72 | 0.70 | 1.03 | 0.72 | 0.71 |
| Insular region | 0.60 | 0.71 | 0.98 | 0.58 | 0.64 |
| Brainstem | 0.78 | 0.71 | 1.08 | 0.79 | 0.77 |
| Frontoparietal region | 0.83 | 0.85 | 1.19 | 0.80 | 0.74 |
| Temporal Region | 0.78 | 0.78 | 1.11 | 0.80 | 0.74 |
| Occipital Lobe | 0.68 | 0.68 | 0.98 | 0.63 | 0.68 |

The table is adopted from: Rashid T, Liu H, Ware J, et al. Deep Learning Based Detection of Enlarged Perivascular Spaces on Brain MRI. 2022 (arXiv:2209.13727v2).
Abbreviations: ePVS, enlarged perivascular spaces.

© 2023 Charisis S,et al. *JAMA Network Open.*

## eTable 2: Associations of regional ePVS volumes with prevalent cardiovascular disease.

| | | All-cause CVD<br>Model 1 | All-cause CVD<br>Model 2 | Stroke<br>Model 1 | Stroke<br>Model 2 | TIA<br>Model 1 | TIA<br>Model 2 |
|---|---|---|---|---|---|---|---|
| Basal Ganglia [-1 * x ^ (-0.2)] | OR | $8.07*10^{1}$ | $2.49*10^{1}$ | $1.95*10^{2}$ | $6.67*10^{1}$ | $2.16*10^{3}$ | $3.33*10^{2}$ |
| | 95% CI | 7.61, $9.01*10^{2}$ | 2.16, $3.00*10^{2}$ | 3.18, $1.33*10^{4}$ | $9.41*10^{-1}$, $5.74*10^{3}$ | 8.83, $8.13*10^{5}$ | 1.20, $1.46*10^{5}$ |
| | p | <0.001 [a] | 0.01 | 0.01 | 0.06 | 0.008 | 0.05 |
| Thalamus [x ^ 0.3] | OR | $4.96*10^{3}$ | $6.08*10^{2}$ | $1.26*10^{2}$ | 6.50 | $4.18*10^{8}$ | $1.74*10^{8}$ |
| | 95% CI | $2.36*10^{1}$, $1.05*10^{6}$ | 2.43, $1.55*10^{5}$ | $1.01*10^{-2}$, $1.20*10^{6}$ | $3.32*10^{-4}$, $1.23*10^{5}$ | $2.78*10^{3}$, $6.74*10^{13}$ | $4.93*10^{2}$, $9.84*10^{13}$ |
| | p | 0.002 [a] | 0.02 | 0.31 | 0.71 | 0.001 [a] | 0.004 [a] |
| Insular region [x ^ 0.35] | OR | $2.08*10^{-1}$ | $3.41*10^{-1}$ | $4.95*10^{1}$ | $3.76*10^{1}$ | $1.57*10^{1}$ | $1.41*10^{1}$ |
| | 95% CI | $1.77*10^{-3}$, $2.36*10^{1}$ | $2.10*10^{-3}$, $5.35*10^{1}$ | $1.07*10^{-2}$, $2.30*10^{5}$ | $6.12*10^{-3}$, $2.44*10^{5}$ | $1.96*10^{-4}$, $9.98*10^{5}$ | $5.55*10^{-5}$, $3.94*10^{6}$ |
| | p | 0.52 | 0.68 | 0.36 | 0.41 | 0.63 | 0.68 |
| Brainstem [x ^ 0.425] | OR | $8.24*10^{1}$ | $3.31*10^{1}$ | $7.81*10^{-2}$ | $2.36*10^{-3}$ | $3.63*10^{7}$ | $2.73*10^{6}$ |
| | 95% CI | $9.87*10^{-5}$, $6.28*10^{7}$ | $2.29*10^{-5}$, $4.32*10^{7}$ | $1.34*10^{-12}$, $2.05*10^{9}$ | $3.55*10^{-14}$, $1.02*10^{8}$ | $2.44*10^{-7}$, $1.13*10^{21}$ | $7.17*10^{-9}$, $1.56*10^{21}$ |
| | p | 0.52 | 0.63 | 0.84 | 0.63 | 0.28 | 0.39 |
| Frontoparietal region [x ^ 0.05] | OR | $1.44*10^{-1}$ | $9.54*10^{-2}$ | $7.13*10^{2}$ | $1.11*10^{1}$ | $7.51*10^{-1}$ | $6.65*10^{-2}$ |
| | 95% CI | $1.59*10^{-5}$, $1.26*10^{3}$ | $7.05*10^{-6}$, $1.26*10^{3}$ | $1.47*10^{-4}$, $3.77*10^{9}$ | $1.95*10^{-6}$, $7.42*10^{7}$ | $3.34*10^{-10}$, $1.35*10^{9}$ | $8.74*10^{-12}$, $5.44*10^{8}$ |
| | p | 0.68 | 0.63 | 0.40 | 0.76 | 0.98 | 0.81 |
| Temporal region [ln(x)] | OR | $9.04*10^{-1}$ | $8.62*10^{-1}$ | 1.28 | 1.05 | 1.20 | 1.16 |
| | 95% CI | $6.38*10^{-1}$, 1.28 | $5.98*10^{-1}$, 1.24 | $7.05*10^{-1}$, 2.32 | $5.68*10^{-1}$, 1.95 | $5.22*10^{-1}$, 2.74 | $4.77*10^{-1}$, 2.90 |
| | p | 0.57 | 0.42 | 0.42 | 0.87 | 0.67 | 0.74 |

Results from generalized linear models with CVD entities as the outcomes, and regional ePVS volumes as predictors.
Model 1 is adjusted for age, sex, ethnicity, field center, and intracranial volume.
Model 2 is further adjusted for systolic blood pressure, use of antihypertensive medications, diabetes, hyperlipidemia, smoking, alcohol consumption, WHR, and physical activity.
Regional ePVS volumes have undergone Tukey’s “ladder of powers” transformation; the respective transformation functions can be found in the brackets.
Values represent odds ratios (OR) for one increment increase in the transformed predictor variable, and their respective 95% confidence intervals (CI) and raw p values.
[a] significant after false discovery rate control at 5% using the Benjamini-Hochberg procedure.
Abbreviations: ePVS, enlarged perivascular spaces; CVD, cardiovascular disease; TIA, transient ischemic attack.

© 2023 Charisis S,et al. *JAMA Network Open.*

## eTable 3: Associations of regional ePVS counts with demographic characteristics.

| | | Age, per year | Sex | | Race/ethnicity | | | |
|---|---|---|---|---|---|---|---|---|
| | | | Women | Men | White, Caucasian | Chinese, American | Black, African-American | Hispanic |
| Basal Ganglia [x ^ 0.175] | β | $2.58*10^{-3}$ | Reference (0) | $-7.47*10^{-3}$ | Reference (0) | $1.41*10^{-2}$ | $-7.74*10^{-3}$ | $2.71*10^{-3}$ |
| | 95% CI | $1.89*10^{-3}$, $3.27*10^{-3}$ | | $-2.24*10^{-2}$, $7.48*10^{-3}$ | | $-5.24*10^{-3}$, $3.35*10^{-2}$ | $-2.29*10^{-2}$, $7.47*10^{-3}$ | $-1.34*10^{-2}$, $1.88*10^{-2}$ |
| | p | <0.001 [a] | | 0.33 | | 0.15 | 0.32 | 0.74 |
| Thalamus [x ^ 0.5] | β | $1.61*10^{-2}$ | Reference (0) | $-1.65*10^{-1}$ | Reference (0) | $-1.57*10^{-1}$ | $8.32*10^{-2}$ | $-1.89*10^{-1}$ |
| | 95% CI | $9.35*10^{-3}$, $2.28*10^{-2}$ | | $-3.11*10^{-1}$, $-1.86*10^{-2}$ | | $-3.46*10^{-1}$, $3.23*10^{-2}$ | $-6.53*10^{-2}$, $2.32*10^{-1}$ | $-3.47*10^{-1}$, $-3.14*10^{-2}$ |
| | p | <0.001 [a] | | 0.03 | | 0.11 | 0.27 | 0.02 |
| Insular region [x ^ 0.5] | β | $6.01*10^{-3}$ | Reference (0) | $-1.05*10^{-1}$ | Reference (0) | $-1.75*10^{-1}$ | $-1.67*10^{-2}$ | $1.09*10^{-1}$ |
| | 95% CI | $-2.68*10^{-3}$, $1.47*10^{-2}$ | | $-2.93*10^{-1}$, $8.27*10^{-2}$ | | $-4.19*10^{-1}$, $6.85*10^{-2}$ | $-2.08*10^{-1}$, $1.75*10^{-1}$ | $-9.43*10^{-2}$, $3.12*10^{-1}$ |
| | p | 0.18 | | 0.27 | | 0.16 | 0.86 | 0.29 |
| Brainstem [x ^ 0.8] | β | $2.16*10^{-3}$ | Reference (0) | $-2.13*10^{-1}$ | Reference (0) | $1.71*10^{-1}$ | $1.74*10^{-1}$ | $-2.72*10^{-1}$ |
| | 95% CI | $-1.27*10^{-2}$, $1.71*10^{-2}$ | | $-5.35*10^{-1}$, $1.10*10^{-1}$ | | $-2.46*10^{-1}$, $5.88*10^{-1}$ | $-1.54*10^{-1}$, $5.01*10^{-1}$ | $-6.19*10^{-1}$, $7.64*10^{-2}$ |
| | p | 0.78 | | 0.20 | | 0.42 | 0.30 | 0.13 |
| Frontoparietal region [x ^ 0.625] | β | $-8.41*10^{-2}$ | Reference (0) | -2.15 | Reference (0) | -3.17 | $-5.07*10^{-1}$ | -1.29 |
| | 95% CI | $-1.61*10^{-1}$, $-7.73*10^{-3}$ | | -3.80, $-4.94*10^{-1}$ | | -5.31, -1.03 | -2.19, 1.17 | -3.07, $4.97*10^{-1}$ |
| | p | 0.03 | | 0.01 | | 0.004 [a] | 0.56 | 0.16 |
| Temporal region [x ^ 0.325] | β | $-1.29*10^{-2}$ | Reference (0) | $-1.90*10^{-1}$ | Reference (0) | $-2.45*10^{-1}$ | $-9.07*10^{-2}$ | $-1.79*10^{-1}$ |
| | 95% CI | $-1.86*10^{-2}$, $-7.08*10^{-3}$ | | $-3.15*10^{-1}$, $-6.54*10^{-2}$ | | $-4.07*10^{-1}$, $-8.33*10^{-2}$ | $-2.18*10^{-1}$, $3.65*10^{-2}$ | $-3.14*10^{-1}$, $-4.45*10^{-2}$ |
| | p | <0.001 [a] | | 0.003 [a] | | 0.003 [a] | 0.16 | 0.009 |

Results from generalized linear models with regional ePVS counts as the outcomes, and demographic characteristics as predictors; models are adjusted for field center, intracranial volume, and vascular risk factors (systolic blood pressure, use of antihypertensive medications, diabetes, hyperlipidemia, smoking, alcohol consumption, WHR, and physical activity).
Regional ePVS counts have undergone Tukey's "ladder of powers" transformation; the respective transformation functions can be found in the brackets.
Values represent beta coefficients (β), reflecting the change in the transformed response variable for one increment increase in the predictor variable, and their respective 95% confidence intervals (CI) and raw p values.
[a] significant after false discovery rate control at 5% using the Benjamini-Hochberg procedure.
[b] systolic blood pressure, use of antihypertensive medications, diabetes, hyperlipidemia, smoking, alcohol consumption, WHR, and physical activity.
Abbreviations: ePVS, enlarged perivascular spaces.

© 2023 Charisis S,et al. *JAMA Network Open.*

**eTable 4: Associations of regional ePVS counts with vascular risk factors.**

| | | SBP, per mmHg | Use of anti-hypertensives | Diabetes | Hyperlipidemia | Smoking | Alcohol | WHR | Physical activity [x ^ 0.375] |
|---|---|---|---|---|---|---|---|---|---|
| Basal Ganglia [x ^ 0.175] | β | $5.17*10^{-4}$ | $2.09*10^{-2}$ | $1.67*10^{-3}$ | $1.10*10^{-2}$ | $1.70*10^{-3}$ | $-5.43*10^{-3}$ | $3.40*10^{-2}$ | $-5.10*10^{-5}$ |
| | 95% CI | $2.41*10^{-4}$, $7.92*10^{-4}$ | $8.88*10^{-3}$, $3.28*10^{-2}$ | $-1.20*10^{-2}$, $1.53*10^{-2}$ | $-5.19*10^{-4}$, $2.25*10^{-2}$ | $-9.25*10^{-3}$, $1.27*10^{-2}$ | $-1.70*10^{-2}$, $6.13*10^{-3}$ | $-4.55*10^{-2}$, $1.14*10^{-1}$ | $-6.74*10^{-4}$, $5.72*10^{-4}$ |
| | p | <0.001 [a] | <0.001 [a] | 0.81 | 0.06 | 0.76 | 0.36 | 0.40 | 0.87 |
| Thalamus [x ^ 0.5] | β | $3.19*10^{-3}$ | $2.48*10^{-1}$ | $-7.46*10^{-2}$ | $4.00*10^{-2}$ | $-3.03*10^{-2}$ | $-1.47*10^{-2}$ | $-1.95*10^{-1}$ | $-6.44*10^{-4}$ |
| | 95% CI | $4.99*10^{-4}$, $5.87*10^{-3}$ | $1.31*10^{-1}$, $3.65*10^{-1}$ | $-2.08*10^{-1}$, $5.85*10^{-2}$ | $-7.23*10^{-2}$, $1.52*10^{-1}$ | $-1.37*10^{-1}$, $7.67*10^{-2}$ | $-1.28*10^{-1}$, $9.83*10^{-2}$ | $-9.71*10^{-1}$, $5.82*10^{-1}$ | $-6.73*10^{-3}$, $5.44*10^{-3}$ |
| | p | 0.02 | <0.001 [a] | 0.27 | 0.49 | 0.58 | 0.80 | 0.62 | 0.84 |
| Insular region [x ^ 0.5] | β | $1.33*10^{-3}$ | $4.91*10^{-3}$ | $1.37*10^{-1}$ | $-1.01*10^{-1}$ | $4.35*10^{-2}$ | $-1.03*10^{-1}$ | $-2.98*10^{-1}$ | $5.55*10^{-3}$ |
| | 95% CI | $-2.13*10^{-3}$, $4.79*10^{-3}$ | $-1.46*10^{-1}$, $1.56*10^{-1}$ | $-3.42*10^{-2}$, $3.09*10^{-1}$ | $-2.45*10^{-1}$, $4.40*10^{-2}$ | $-9.43*10^{-2}$, $1.81*10^{-1}$ | $-2.49*10^{-1}$, $4.23*10^{-2}$ | -1.30, $7.02*10^{-1}$ | $-2.28*10^{-3}$, $1.34*10^{-2}$ |
| | p | 0.45 | 0.95 | 0.12 | 0.17 | 0.54 | 0.17 | 0.56 | 0.17 |
| Brainstem [x ^ 0.8] | β | $5.63*10^{-4}$ | $2.21*10^{-1}$ | $-1.37*10^{-1}$ | $-9.03*10^{-2}$ | $8.50*10^{-2}$ | $7.33*10^{-2}$ | -2.43 | $-4.05*10^{-3}$ |
| | 95% CI | $-5.37*10^{-3}$, $6.49*10^{-3}$ | $-3.69*10^{-2}$, $4.80*10^{-1}$ | $-4.31*10^{-1}$, $1.57*10^{-1}$ | $-3.38*10^{-1}$, $1.58*10^{-1}$ | $-1.51*10^{-1}$, $3.21*10^{-1}$ | $-1.76*10^{-1}$, $3.23*10^{-1}$ | -4.15, $-7.17*10^{-1}$ | $-1.75*10^{-2}$, $9.38*10^{-3}$ |
| | p | 0.85 | 0.09 | 0.36 | 0.48 | 0.48 | 0.56 | 0.006 [a] | 0.56 |
| Frontoparietal region [x ^ 0.625] | β | $4.47*10^{-2}$ | $6.80*10^{-1}$ | 1.47 | -1.10 | $-6.19*10^{-1}$ | $-1.13*10^{-1}$ | $-1.06*10^{1}$ | $-2.95*10^{-2}$ |
| | 95% CI | $1.42*10^{-2}$, $7.51*10^{-2}$ | $-6.44*10^{-1}$, 2.01 | $-3.52*10^{-2}$, 2.98 | -2.37, $1.75*10^{-1}$ | -1.83, $5.92*10^{-1}$ | -1.39, 1.17 | $-1.94*10^{1}$, -1.85 | $-9.84*10^{-2}$, $3.94*10^{-2}$ |
| | p | 0.004 [a] | 0.31 | 0.06 | 0.09 | 0.32 | 0.86 | 0.02 | 0.40 |
| Temporal region [x ^ 0.325] | β | $2.89*10^{-3}$ | $7.92*10^{-2}$ | $1.32*10^{-1}$ | $-7.10*10^{-2}$ | $-7.28*10^{-2}$ | $8.03*10^{-3}$ | $-6.10*10^{-1}$ | $-6.55*10^{-4}$ |
| | 95% CI | $5.93*10^{-4}$, $5.20*10^{-3}$ | $-2.10*10^{-2}$, $1.79*10^{-1}$ | $1.75*10^{-2}$, $2.46*10^{-1}$ | $-1.67*10^{-1}$, $2.52*10^{-2}$ | $-1.64*10^{-1}$, $1.88*10^{-2}$ | $-8.87*10^{-2}$, $1.05*10^{-1}$ | -1.27, $5.51*10^{-2}$ | $-5.86*10^{-3}$, $4.55*10^{-3}$ |
| | p | 0.01 | 0.12 | 0.02 | 0.15 | 0.12 | 0.87 | 0.07 | 0.81 |

Results from generalized linear models with regional ePVS counts as the outcomes, and vascular risk factors as predictors; models are adjusted for age, sex, ethnicity, field center, and intracranial volume.
Regional ePVS counts and intentional physical activity (in MET/min/week) have undergone Tukey's "ladder of powers" transformation; the respective transformation functions can be found in the brackets.
Values represent beta coefficients (β), reflecting the change in the transformed response variable for one increment increase in the predictor variable, and their respective 95% confidence intervals (CI) and raw p values.
[a] significant after false discovery rate control at 5% using the Benjamini-Hochberg procedure.
Abbreviations: ePVS, enlarged perivascular spaces; SBP, systolic blood pressure; WHR, waist-to-hip ratio; MET, metabolic equivalent of task.

© 2023 Charisis S,et al. *JAMA Network Open.*

## eTable 5: Associations of regional ePVS counts with structural MRI indices.

| | | Total Grey Matter Volume | Total WMH volume [x ^ 0.025] | Presence of CMBs (OR) | Total WM Fractional Anisotropy |
|---|---|---|---|---|---|
| Basal Ganglia [x ^ 0.175] | β | $-9.36*10^{3}$ | $4.19*10^{-1}$ | $1.10*10^{1}$ | $-5.91*10^{-2}$ |
| | 95% CI | $-2.98*10^{4}$, $1.10*10^{4}$ | $3.63*10^{-1}$, $4.76*10^{-1}$ | 2.33, $5.27*10^{1}$ | $-7.44*10^{-2}$, $-4.37*10^{-2}$ |
| | p | 0.37 | <0.001 [a] | 0.003 [a] | <0.001 [a] |
| Thalamus [x ^ 0.5] | β | $-3.58*10^{2}$ | $2.87*10^{-2}$ | 1.30 | $-4.68*10^{-3}$ |
| | 95% CI | $-2.46*10^{3}$, $1.75*10^{3}$ | $2.25*10^{-2}$, $3.49*10^{-2}$ | 1.10, 1.52 | $-6.29*10^{-3}$, $-3.08*10^{-3}$ |
| | p | 0.74 | <0.001 [a] | 0.002 [a] | <0.001 [a] |
| Insular region [x ^ 0.5] | β | $6.88*10^{2}$ | $-1.04*10^{-2}$ | $9.98*10^{-1}$ | $-4.10*10^{-4}$ |
| | 95% CI | $-9.64*10^{2}$, $2.34*10^{3}$ | $-1.54*10^{-2}$, $-5.43*10^{-3}$ | $8.80*10^{-1}$, 1.13 | $-1.70*10^{-3}$, $8.76*10^{-4}$ |
| | p | 0.41 | <0.001 [a] | 0.97 | 0.53 |
| Brainstem [x ^ 0.8] | β | $-4.98*10^{1}$ | $-1.77*10^{-3}$ | 1.06 | $-1.76*10^{-4}$ |
| | 95% CI | $-1.01*10^{3}$, $9.13*10^{2}$ | $-4.72*10^{-3}$, $1.18*10^{-3}$ | $9.86*10^{-1}$, 1.14 | $-9.24*10^{-4}$, $5.71*10^{-4}$ |
| | p | 0.92 | 0.24 | 0.12 | 0.64 |
| Frontoparietal region [x ^ 0.625] | β | $8.67*10^{1}$ | $-2.56*10^{-4}$ | 1.01 | $-1.37*10^{-4}$ |
| | 95% CI | $-9.99*10^{1}$, $2.73*10^{2}$ | $-8.24*10^{-4}$, $3.13*10^{-4}$ | $9.97*10^{-1}$, 1.03 | $-2.81*10^{-4}$, $7.98*10^{-6}$ |
| | p | 0.36 | 0.38 | 0.11 | 0.06 |
| Temporal region [x ^ 0.325] | β | $2.89*10^{3}$ | $2.23*10^{-3}$ | 1.13 | $-3.01*10^{-3}$ |
| | 95% CI | $4.27*10^{2}$, $5.35*10^{3}$ | $-5.30*10^{-3}$, $9.76*10^{-3}$ | $9.41*10^{-1}$, 1.37 | $-4.92*10^{-3}$, $-1.10*10^{-3}$ |
| | p | 0.02 | 0.56 | 0.19 | 0.002 [a] |

Results from generalized linear models with structural MRI indices as the outcomes, and regional ePVS counts as predictors; models are adjusted for age, sex, ethnicity, field center, and intracranial volume.
Regional ePVS counts and total WMH volume have undergone Tukey's "ladder of powers" transformation; the respective transformation functions can be found in the brackets.
Values represent beta coefficients (β), reflecting the change in the response variable for one increment increase in the transformed predictor variable, and their respective 95% confidence intervals (CI) and raw p values. For CMBs odds ratios (OR) are presented instead of beta coefficients.
[a] significant after false discovery rate control at 5% using the Benjamini-Hochberg procedure.
Abbreviations: ePVS, enlarged perivascular spaces; WMH, white matter hyperintensity; CMBs, cerebral microbleeds; WM, white matter.

© 2023 Charisis S,et al. *JAMA Network Open.*

**eTable 6: Associations of regional ePVS counts with prevalent cardiovascular disease.**

| | | All-cause CVD Model 1 | All-cause CVD Model 2 | Stroke Model 1 | Stroke Model 2 | TIA Model 1 | TIA Model 2 |
|---|---|---|---|---|---|---|---|
| Basal Ganglia [x ^ 0.175] | OR | $4.02*10^{2}$ | $1.39*10^{2}$ | $2.88*10^{2}$ | $1.07*10^{2}$ | $1.18*10^{2}$ | $1.95*10^{1}$ |
| | 95% CI | $2.37*10^{1}$, $7.29*10^{3}$ | 7.22, $2.85*10^{3}$ | 2.61, $3.55*10^{4}$ | $8.39*10^{-1}$, $1.53*10^{4}$ | $2.10*10^{-1}$, $7.37*10^{4}$ | $2.74*10^{-2}$, $1.53*10^{4}$ |
| | p | <0.001 [a] | 0.001 [a] | 0.02 | 0.06 | 0.14 | 0.37 |
| Thalamus [x ^ 0.5] | OR | 1.60 | 1.42 | 1.76 | 1.53 | 2.89 | 2.43 |
| | 95% CI | 1.22, 2.11 | 1.07, 1.88 | 1.10, 2.84 | $9.47*10^{-1}$, 2.51 | 1.53, 5.60 | 1.27, 4.84 |
| | p | <0.001 [a] | 0.01 | 0.02 | 0.09 | 0.001 [a] | 0.009 |
| Insular region [x ^ 0.5] | OR | 1.04 | 1.07 | 1.49 | 1.53 | $8.86*10^{-1}$ | $8.60*10^{-1}$ |
| | 95% CI | $8.35*10^{-1}$, 1.28 | $8.53*10^{-1}$, 1.35 | 1.03, 2.17 | 1.04, 2.27 | $5.24*10^{-1}$, 1.48 | $4.79*10^{-1}$, 1.52 |
| | p | 0.75 | 0.55 | 0.03 | 0.03 | 0.65 | 0.61 |
| Brainstem [x ^ 0.8] | OR | 1.13 | 1.13 | 1.14 | 1.14 | $9.82*10^{-1}$ | $9.60*10^{-1}$ |
| | 95% CI | $9.97*10^{-1}$, 1.29 | $9.91*10^{-1}$, 1.29 | $9.13*10^{-1}$, 1.43 | $9.00*10^{-1}$, 1.45 | $7.28*10^{-1}$, 1.32 | $7.10*10^{-1}$, 1.31 |
| | p | 0.06 | 0.07 | 0.25 | 0.29 | 0.90 | 0.79 |
| Frontoparietal region [x ^ 0.625] | OR | $9.99*10^{-1}$ | $9.96*10^{-1}$ | 1.02 | 1.01 | 1.01 | $9.96*10^{-1}$ |
| | 95% CI | $9.75*10^{-1}$, 1.02 | $9.72*10^{-1}$, 1.02 | $9.84*10^{-1}$, 1.07 | $9.70*10^{-1}$, 1.06 | $9.50*10^{-1}$, 1.07 | $9.39*10^{-1}$, 1.06 |
| | p | 0.94 | 0.78 | 0.25 | 0.62 | 0.85 | 0.91 |
| Temporal region [x ^ 0.325] | OR | $9.64*10^{-1}$ | $9.29*10^{-1}$ | 1.39 | 1.17 | 1.05 | $9.53*10^{-1}$ |
| | 95% CI | $7.03*10^{-1}$, 1.32 | $6.68*10^{-1}$, 1.29 | $8.09*10^{-1}$, 2.39 | $6.76*10^{-1}$, 2.07 | $4.87*10^{-1}$, 2.24 | $4.27*10^{-1}$, 2.17 |
| | p | 0.82 | 0.66 | 0.23 | 0.57 | 0.91 | 0.91 |

Results from generalized linear models with CVD entities as the outcomes, and regional ePVS counts as predictors.
Model 1 is adjusted for age, sex, ethnicity, field center, and intracranial volume.
Model 2 is further adjusted for systolic blood pressure, use of antihypertensive medications, diabetes, hyperlipidemia, smoking, alcohol consumption, WHR, and physical activity.
Regional ePVS counts have undergone Tukey's "ladder of powers" transformation; the respective transformation functions can be found in the brackets.
Values represent odds ratios (OR) for one increment increase in the transformed predictor variable, and their respective 95% confidence intervals (CI) and raw p values.
[a] significant after false discovery rate control at 5% using the Benjamini-Hochberg procedure.
Abbreviations: ePVS, enlarged perivascular spaces; CVD, cardiovascular disease; TIA, transient ischemic attack.

© 2023 Charisis S,et al. *JAMA Network Open.*

| eTable 7: Associations of regional ePVS volumes with vascular risk factors for low regional ePVS volumes (at the 25th percentile). | | | | | | | | | |
|---|---|---|---|---|---|---|---|---|---|
| | | SBP, per mmHg | Use of anti-hypertensives | Diabetes | Hyperlipidemia | Smoking | Alcohol | WHR | Physical activity [x ^ 0.375] |
| Basal Ganglia | β | 1.41 | 29.65 | -14.07 | 11.26 | -4.53 | -9.35 | 174.58 | 0.43 |
| | SE | 0.35 | 16.44 | 21.61 | 15.88 | 14.51 | 13.91 | 94.51 | 0.77 |
| | p | <0.001 [a] | 0.07 | 0.52 | 0.48 | 0.75 | 0.50 | 0.07 | 0.58 |
| Thalamus | β | 0.06 | 4.73 | -2.22 | 0.33 | -0.13 | 1.86 | -6.92 | -0.07 |
| | SE | 0.04 | 1.54 | 1.66 | 1.58 | 1.47 | 1.52 | 10.68 | 0.08 |
| | p | 0.10 | 0.002 [a] | 0.18 | 0.83 | 0.93 | 0.22 | 0.52 | 0.38 |
| Insular region | β | -0.01 | 0.63 | 0.91 | -1.51 | -0.36 | -0.44 | -1.67 | -0.02 |
| | SE | 0.02 | 0.71 | 1.03 | 0.75 | 0.66 | 0.69 | 4.28 | 0.04 |
| | p | 0.48 | 0.38 | 0.38 | 0.04 | 0.59 | 0.53 | 0.70 | 0.55 |
| Brainstem | β | -0.03 | 2.75 | -2.91 | -0.86 | -0.98 | 2.27 | -27.91 | 0.05 |
| | SE | 0.06 | 2.31 | 2.32 | 2.22 | 2.11 | 2.18 | 15.73 | 0.12 |
| | p | 0.61 | 0.23 | 0.21 | 0.70 | 0.64 | 0.30 | 0.08 | 0.67 |
| Frontoparietal region | β | 2.76 | 36.97 | 100.02 | -101.71 | -62.29 | -0.54 | -415.66 | -6.91 |
| | SE | 1.39 | 64.02 | 73.68 | 60.47 | 62.82 | 60.81 | 447.80 | 3.40 |
| | p | 0.05 | 0.56 | 0.17 | 0.09 | 0.32 | 0.99 | 0.35 | 0.04 |
| Temporal region | β | 0.66 | 25.29 | 15.16 | -1.70 | -19.93 | -0.08 | -198.56 | -0.98 |
| | SE | 0.29 | 11.79 | 15.67 | 13.77 | 12.39 | 12.16 | 78.56 | 0.67 |
| | p | 0.02 | 0.03 | 0.33 | 0.90 | 0.11 | 0.99 | 0.01 | 0.15 |

Results from quantile regression models at the 0.25 quantile with regional ePVS volumes as the outcomes, and vascular risk factors as predictors; models are adjusted for age, sex, ethnicity, field center, and total intracranial volume.
Intentional physical activity (in MET/min/week) has undergone Tukey's "ladder of powers" transformation; the respective transformation function can be found in the brackets.
Values represent beta coefficients (β), reflecting the change in the response variable for one increment increase in the predictor variable, and their respective standard errors (SE) and raw p values.
[a] significant after false discovery rate control at 5% using the Benjamini-Hochberg procedure.
Abbreviations: ePVS, enlarged perivascular spaces; SBP, systolic blood pressure; WHR, waist-to-hip ratio; MET, metabolic equivalent of task.

© 2023 Charisis S,et al. *JAMA Network Open.*

**eTable 8: Associations of regional ePVS volumes with vascular risk factors for median regional ePVS volumes (at the 50th percentile).**

| | | SBP, per mmHg | Use of anti-hypertensives | Diabetes | Hyperlipidemia | Smoking | Alcohol | WHR | Physical activity [x ^ 0.375] |
|---|---|---|---|---|---|---|---|---|---|
| Basal Ganglia | β | 2.63 | 74.00 | -23.88 | 17.37 | -13.79 | -25.02 | 275.66 | -0.43 |
| | SE | 0.45 | 17.11 | 23.33 | 18.86 | 14.62 | 15.77 | 110.80 | 0.86 |
| | p | <0.001 [a] | <0.001 [a] | 0.31 | 0.36 | 0.35 | 0.11 | 0.01 | 0.62 |
| Thalamus | β | 0.14 | 5.59 | -5.03 | 2.97 | -5.95 | 1.68 | -27.63 | -0.09 |
| | SE | 0.06 | 2.64 | 3.27 | 2.47 | 2.20 | 2.46 | 15.77 | 0.12 |
| | p | 0.03 | 0.03 | 0.12 | 0.23 | 0.007 | 0.50 | 0.08 | 0.46 |
| Insular region | β | 0.02 | -0.21 | 3.25 | -2.13 | 0.97 | -1.40 | -4.68 | 0.06 |
| | SE | 0.03 | 1.32 | 1.80 | 1.28 | 1.24 | 1.25 | 8.17 | 0.07 |
| | p | 0.48 | 0.88 | 0.07 | 0.10 | 0.43 | 0.27 | 0.57 | 0.38 |
| Brainstem | β | 0.08 | 4.61 | -6.13 | -1.91 | -1.30 | 1.06 | -28.81 | -0.02 |
| | SE | 0.05 | 2.10 | 2.65 | 2.02 | 2.03 | 2.09 | 13.74 | 0.10 |
| | p | 0.09 | 0.03 | 0.02 | 0.34 | 0.52 | 0.61 | 0.04 | 0.80 |
| Frontoparietal region | β | 4.46 | 125.22 | 193.84 | -84.86 | -16.47 | 80.49 | -1571.42 | -1.19 |
| | SE | 2.11 | 80.99 | 100.74 | 79.85 | 79.70 | 83.03 | 591.10 | 4.27 |
| | p | 0.04 | 0.12 | 0.05 | 0.29 | 0.84 | 0.33 | 0.008 | 0.78 |
| Temporal region | β | 1.64 | 28.25 | 51.17 | 15.03 | -20.13 | 12.59 | -313.32 | -1.63 |
| | SE | 0.61 | 24.34 | 35.34 | 22.79 | 23.13 | 23.09 | 162.49 | 1.34 |
| | p | 0.007 | 0.25 | 0.15 | 0.51 | 0.38 | 0.59 | 0.05 | 0.22 |

Results from quantile regression models at the 0.50 quantile with regional ePVS volumes as the outcomes, and vascular risk factors as predictors; models are adjusted for age, sex, ethnicity, field center, and total intracranial volume.
Intentional physical activity (in MET/min/week) has undergone Tukey's "ladder of powers" transformation; the respective transformation function can be found in the brackets.
Values represent beta coefficients (β), reflecting the change in the response variable for one increment increase in the predictor variable, and their respective standard errors (SE) and raw p values.
[a] significant after false discovery rate control at 5% using the Benjamini-Hochberg procedure.
Abbreviations: ePVS, enlarged perivascular spaces; SBP, systolic blood pressure; WHR, waist-to-hip ratio; MET, metabolic equivalent of task.

© 2023 Charisis S,et al. *JAMA Network Open.*

**eTable 9: Associations of regional ePVS volumes with vascular risk factors for high regional ePVS volumes (at the 75th percentile).**

| | | SBP, per mmHg | Use of anti-hypertensives | Diabetes | Hyperlipidemia | Smoking | Alcohol | WHR | Physical activity [x ^ 0.375] |
|---|---|---|---|---|---|---|---|---|---|
| Basal Ganglia | β | 3.28 | 132.76 | -41.64 | 45.20 | -24.24 | -63.77 | 413.03 | -1.07 |
| | SE | 0.89 | 34.84 | 47.63 | 36.03 | 31.43 | 33.38 | 232.47 | 1.77 |
| | p | <0.001 [a] | <0.001 [a] | 0.38 | 0.21 | 0.44 | 0.06 | 0.08 | 0.55 |
| Thalamus | β | 0.30 | 11.37 | -3.62 | -1.12 | -4.04 | 1.13 | -37.59 | 0.03 |
| | SE | 0.10 | 4.37 | 4.46 | 4.20 | 3.95 | 4.23 | 28.87 | 0.22 |
| | p | 0.001 [a] | 0.009 [a] | 0.42 | 0.79 | 0.31 | 0.79 | 0.19 | 0.89 |
| Insular region | β | 0.03 | 1.08 | 3.68 | -3.16 | -0.95 | -2.18 | -4.25 | 0.05 |
| | SE | 0.04 | 1.97 | 2.89 | 1.83 | 1.92 | 2.00 | 7.85 | 0.11 |
| | p | 0.47 | 0.58 | 0.20 | 0.08 | 0.62 | 0.28 | 0.59 | 0.67 |
| Brainstem | β | 0.08 | 8.09 | -1.17 | -0.15 | -1.74 | -1.09 | -20.04 | -0.18 |
| | SE | 0.08 | 3.44 | 5.06 | 3.29 | 3.15 | 3.35 | 23.29 | 0.18 |
| | p | 0.35 | 0.02 | 0.82 | 0.96 | 0.58 | 0.74 | 0.39 | 0.33 |
| Frontoparietal region | β | 9.40 | 166.65 | 443.74 | -160.90 | -162.18 | -6.36 | -1209.67 | 3.46 |
| | SE | 3.33 | 138.60 | 145.47 | 116.00 | 112.57 | 118.81 | 843.40 | 6.55 |
| | p | 0.005 [a] | 0.23 | 0.002 [a] | 0.17 | 0.15 | 0.96 | 0.15 | 0.60 |
| Temporal region | β | 1.83 | 72.14 | 128.76 | -6.98 | -47.43 | -34.44 | -647.69 | 4.08 |
| | SE | 0.96 | 37.03 | 43.30 | 36.81 | 37.13 | 34.77 | 240.71 | 2.02 |
| | p | 0.06 | 0.05 | 0.003 [a] | 0.85 | 0.20 | 0.32 | 0.007 [a] | 0.04 |

Results from quantile regression models at the 0.75 quantile with regional ePVS volumes as the outcomes, and vascular risk factors as predictors; models are adjusted for age, sex, ethnicity, field center, and total intracranial volume.
Intentional physical activity (in MET/min/week) has undergone Tukey's "ladder of powers" transformation; the respective transformation function can be found in the brackets.
Values represent beta coefficients (β), reflecting the change in the response variable for one increment increase in the predictor variable, and their respective standard errors (SE) and raw p values.
[a] significant after false discovery rate control at 5% using the Benjamini-Hochberg procedure.
Abbreviations: ePVS, enlarged perivascular spaces; SBP, systolic blood pressure; WHR, waist-to-hip ratio; MET, metabolic equivalent of task.

© 2023 Charisis S,et al. *JAMA Network Open.*

**eFigure 1: Flowchart of study sample.**

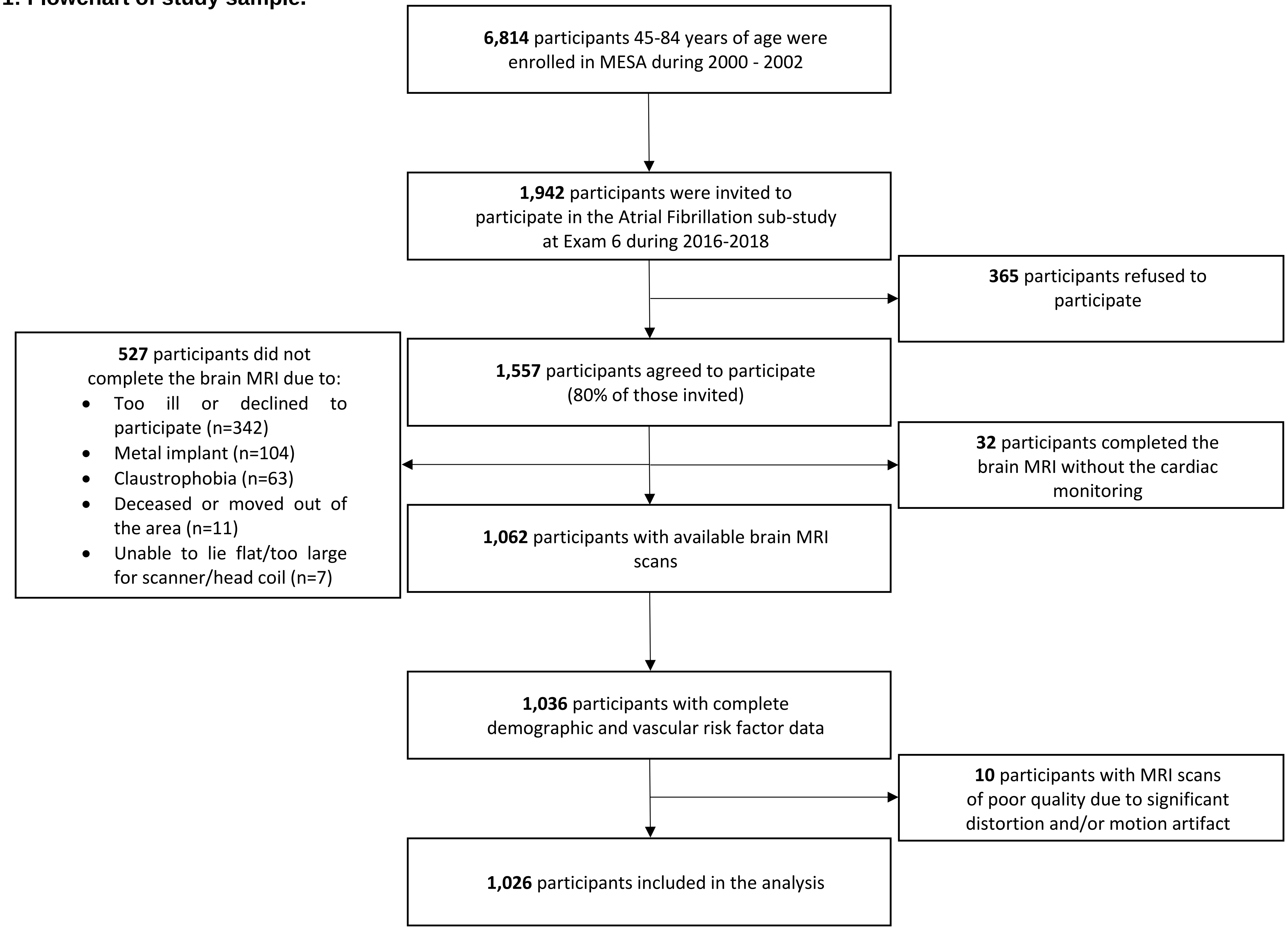


© 2023 Charisis S,et al. *JAMA Network Open.*

**eFigure 2: Anatomic regions based on the existing MUSE segmentations.**

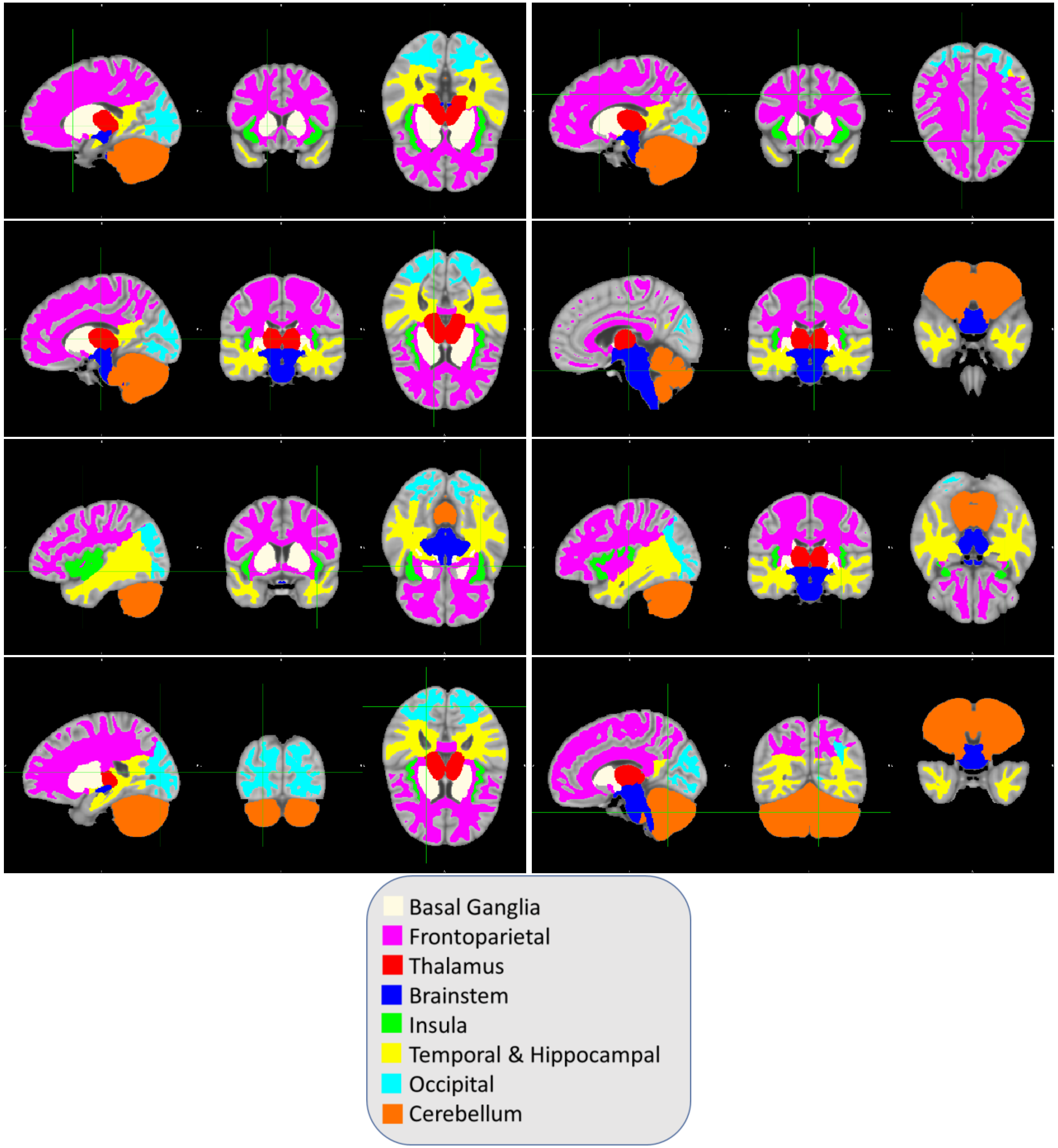


© 2023 Charisis S,et al. *JAMA Network Open.*

**eFigure 3: Predicted normalized regional ePVS volumes (y-axis) over age (x-axis). The plot is derived from adjusted (for sex, ethnicity, field center, intracranial volume, and vascular risk factors) Generalized Linear Models. Prediction was performed with covariates set at their mean values and factors set at their reference levels. Shaded areas represent 95% confidence intervals for the respective normalized regional ePVS volumes.**

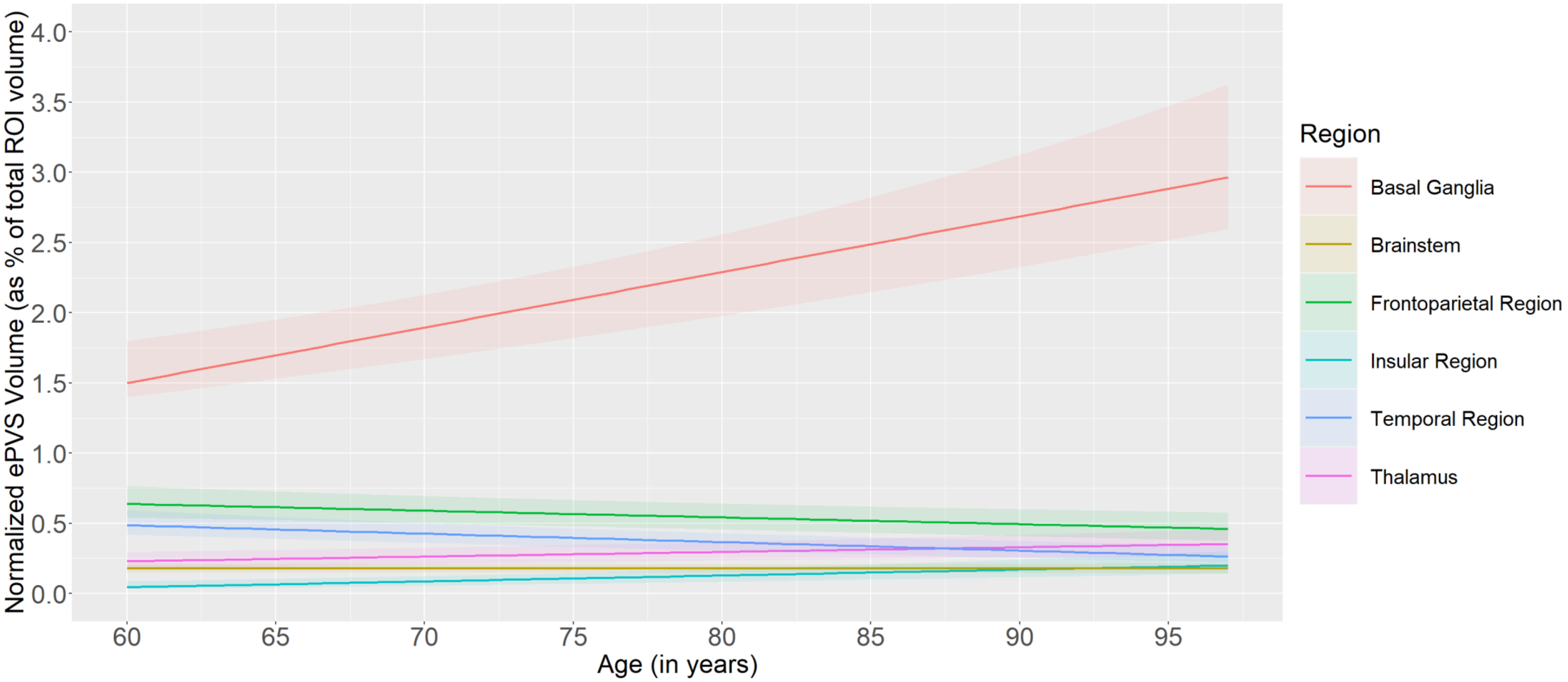


© 2023 Charisis S,et al. *JAMA Network Open.*
