# Supplementary material for: Assessment of Risk Factors and Clinical Importance of Enlarged Perivascular Spaces by Whole-Brain Investigation in the Multi-Ethnic Study of Atherosclerosis
Source: JAMA Netw Open. 2023 Apr 24;6(4):e239196. doi: 10.1001/jamanetworkopen.2023.9196 (PMC10126873; doi:10.1001/jamanetworkopen.2023.9196)
Supplement: Supplement 2. — Data Sharing Statement [file jamanetwopen-e239196-s002.pdf]

Data Sharing Statement

Charisis. Assessment of Risk Factors and Clinical Importance of Enlarged Perivascular Spaces by Whole-Brain Investigation in the Multi-Ethnic Study of Atherosclerosis. *JAMA Netw Open*. Published April 24, 2023. doi:10.1001/jamanetworkopen.2023.9196

Data
Data available: No

Additional Information
Explanation for why data not available: The data that support the findings of this study are available from the corresponding author (Mohamad Habes), upon reasonable request. Request can be sent to: Mohamad Habes, PhD 7703 Floyd Curl Drive San Antonio TX 78229 Email: habes@uthscsa.edu Phone: +1 210-450-8416
